# Fate of β-Carotene within Loaded Delivery Systems in Food: State of Knowledge

**DOI:** 10.3390/antiox10030426

**Published:** 2021-03-10

**Authors:** Vaibhav Kumar Maurya, Amita Shakya, Manjeet Aggarwal, Kodiveri Muthukaliannan Gothandam, Torsten Bohn, Sunil Pareek

**Affiliations:** 1 Department of Basic and Applied Science, National Institute of Food Technology Entrepreneurship and Management, Kundli, Sonepat 131 028, Haryana, India; vaibhavmaurya.niftem@gmail.com (V.K.M.); aggarwal.manjeet@gmail.com (M.A.); 2 Department of Agriculture and Environmental Sciences, National Institute of Food Technology Entrepreneurship and Management, Kundli, Sonepat 131 028, Haryana, India; amitashakya88@gmail.com; 3 School of Bio Sciences and Technology, VIT University, Vellore 632 014, Tamil Nadu, India; gothandam@yahoo.com; 4 Nutrition and Health Research Group, Department of Population Health, Luxembourg Institute of Health, L-1445 Strassen, Luxembourg; Torsten.Bohn@lih.lu

**Keywords:** beta-carotene, bioavailability, delivery system, encapsulation, engineered nanomaterial, SLNs, NLCs

## Abstract

Nanotechnology has opened new opportunities for delivering bioactive agents. Their physiochemical characteristics, i.e., small size, high surface area, unique composition, biocompatibility and biodegradability, make these nanomaterials an attractive tool for β-carotene delivery. Delivering β-carotene through nanoparticles does not only improve its bioavailability/bioaccumulation in target tissues, but also lessens its sensitivity against environmental factors during processing. Regardless of these benefits, nanocarriers have some limitations, such as variations in sensory quality, modification of the food matrix, increasing costs, as well as limited consumer acceptance and regulatory challenges. This research area has rapidly evolved, with a plethora of innovative nanoengineered materials now being in use, including micelles, nano/microemulsions, liposomes, niosomes, solidlipid nanoparticles, nanostructured lipids and nanostructured carriers. These nanodelivery systems make conventional delivery systems appear archaic and promise better solubilization, protection during processing, improved shelf-life, higher bioavailability as well as controlled and targeted release. This review provides information on the state of knowledge on β-carotene nanodelivery systems adopted for developing functional foods, depicting their classifications, compositions, preparation methods, challenges, release and absorption of β-carotene in the gastrointestinal tract (GIT) and possible risks and future prospects.

## 1. Introduction

Vitamin A deficiency is one of the most diagnosed micronutrient deficiency disorders worldwide, especially in developing countries. However, its magnitude is more widespread in the vegetarian population [1]. Across the globe, approximately 250 million preschool children are estimated to be affected by vitamin A deficiency [2]. Furthermore, occurrence of disease has an intimate relationship with a low antioxidant load in the daily diet. Furthermore, lifestyle (exercise, smoking, drinking and high consumption of meat-based and processed foods), environment (emotional and social stress), and cultural constraints trigger the expression of housekeeping genes to adopting genes to retain the cellular, organ or body homeostasis [3]. The aforesaid stimuli also cause the generation of reactive oxygen species (ROS), resulting in oxidative homoeostasis imbalance at cellular and tissue levels, thus generating oxidative stress [4]. Oxidative stress can be defined as a phenomenon triggered by an imbalance between the generation and accumulation of ROS. In general, ROS, including organic hydro peroxides, hydrogen peroxide, nitric oxide, hydroxyl radicals and superoxide, are generated as by-products of oxygen metabolism; in addition, these environmental stimuli (UV, pollutants, heavy metals, and xenobiotics (including antiblastic drugs, antiallergic drugs, immunosuppressant drugs) equally contribute to ROS production, thus causing oxidative stress [5]. Accruing scientific evidence is accumulating on the involvement of oxidative stress in the occurrence of several health complications, which are attributed to inactivation of metabolic enzymes and damage vital cellular components, oxidization the nucleic acids, resulting in eye disorders, atherosclerosis, cardiovascular diseases, joint and bone disorders, neurological diseases (amyotrophic lateral sclerosis, Parkinson’s disease and Alzheimer’s disease) and misfunctioning of different organ including lung, kidney, liver and reproductive system [6]. ROS are primarily generated in mitochondria under both pathological as well as physiological conditions [7]. Cells activate an antioxidant defensive system which primarily includes enzymatic components such as superoxide dismutase, glutathione peroxidase, and catalase in order to minimize the oxidative stress cell [8].

### 1.1. Oxidative Stress and Antioxidants

ROS generation is attributed to both nonenzymatic and enzymatic reactions. Enzymatic processes that have intricate involvement in the respiratory chain, phagocytosis, prostaglandins biosynthesis, and cytochrome P450 system are responsible for ROS generation. Superoxide radicals produced as the result of enzymatic action of NADPH oxidase, peroxidases and xanthine oxidase initiate the chain reaction for ROS formation including hydrogen peroxide, hydroxyl radicals, peroxynitrite, hypochlorous acid and so on [9]. Hydroxyl radicals (^•^OH) are considered as the most reactive among all ROS in vivo and are produced as a result of catalysis of H_2_O_2_ in the presence of Fe^2+^ or Cu^+^ (Fenton reactions).

In addition, some nonenzymatic processes also contribute to ROS generation, especially when oxygen is either exposed to ionizing radiations or reacts with organic compounds. ROS are produced due to exogenous and endogenous sources. Exogenous sources of ROS include inflammation, immune cell activation, infection, ischemia, cancer, mental stress, excessive exercise and aging [4,10]. Exogeneous ROS generation relies on exposure to radiation, heavy metals [11], environmental pollutants [12], certain drugs (bleomycin, cyclosporine, gentamycin, tacrolimus) [13], toxic chemical and solvents [13], food processing (used oil and fat and smoked meat) [14], cigarette smoking and alcohol consumption, among other [10]. ROS are essential part of several biological processes when they remain at low or moderate concentrations. For instance, these ROS are obligatory for synthesis of some cellular structures, which have vital role in the host defense system, i.e. in the defence of pathogens [14,15]. In fact, macrophages synthesize and store ROS to kill pathogenic microbes [16]. The critical role of ROS in the immune system is well recognized as patients unable to produce ROS are more prone to pathological infections [17]. In addition, ROS are also integrated in an array of cellular signaling pathways as they play a regulatory role in intracellular signaling cascades, including endothelial cells, fibroblasts, cardiac myocytes, vascular smooth muscle cells and thyroid tissue. Nitric oxide (NO) is considered as a key cell-to-cell messenger, which plays a vital role in cell signaling and is intricately involved in several processes, such as blood flow modulation, thrombosis and normal neural functioning [18]. Nitric oxide also demonstrates close association with nonspecific host defense in eliminating the tumor cells, as well as intracellular pathogens [19]. In addition to beneficial effects, ROS also pose several negative impacts by affecting cellular structure, including plasma membrane, proteins, lipoprotein, proteins and nucleic acids (deoxyribonucleic acid, DNA; ribonucleic acid, RNA). Oxidative stress is a result of ROS imbalance between its rate of generation and rate of clearance within the cell [20]. These excess ROS thus cause damage in the plasma membrane by lipid peroxidation and form malondialdehyde and conjugated dienes which are cytotoxic and mutagenic in nature. Being a chain reaction cascade, lipid peroxidation spreads very rapidly, damaging a significant number of lipids, proteins and nucleic acids, hence hampering their functionalities [21]. In summary, ROS impart beneficial effects when they are maintained at low or moderate concentrations while they negatively affect several cellular structures at higher concentrations.

The human body adopts several strategies to combat the negative effects generated due to oxidative stress, including enzymatic (superoxide dismutase, glutathione peroxidase and catalase) or nonenzymatic (L-arginine, glutathione, coenzyme Q10 and lipoic acid) antioxidant molecules. In addition to the aforesaid molecules, several exogenous antioxidants molecules from animal or plant origins are deliberately incorporated, i.e. fortified, into the diet [5].

### 1.2. Mode of Action of β-Carotene against Oxidative Stress

β-Carotene, a key member of the carotenoid family, is recognized as one of the most potent antioxidants [22] and the major provitamin A carotenoid available in the human diet. The health benefits of β-carotene are attributed to its given biological properties [21]: (a) as antioxidants that scavenge and quench ROS of oxidative metabolism, (b) as provitamin A compounds that activate retinol-mediated pathways, (c) as electrophiles that boost endogenous antioxidant systems, (d) by hampering inflammation-related processes mediated by nuclear factor κ-light-chain-enhancer of activated B cell (NF-κB) pathway, and/or (e) by directly binding nuclear receptors (NRs) and other transcription factors in target cells.

Retinoic acid acts as ligand for the retinoid X receptors (RXRs) and canonical retinoid acid receptors (RARs), which influence the expression of a number of responsive genes and have intimate relationships with fatty acid, cholesterol, Ca^2+^ and phosphate homeostasis [23]. β-Carotene also demonstrated tumor cell suppression activity and enhanced intercellular communication at gap junctions [3]. It is believed that consumption of β-carotene may cause low incidence of hepatic oxidative stress and lipid oxidation. The assumption was supported by a mice model study where expression of 1207 genes (approximately 4% genes) of a total of 30,855 genes in a hepatic transcriptome was influenced when mice were fed with β-carotene as compared to control mice [24]. Remarkably, numerous differentially expressed genes were intimately involved in energy metabolism, lipid metabolism, and mitochondrial redox homeostasis.

β-Carotene is the main contributor to vitamin A in human beings, if preformed vitamin A intake is insufficient. It acts as a precursor of vitamin A, with the potential to yield two retinal molecules following cleavage by beta-carotene oxygenase 1 in the intestine, as compared to other carotenoids which generally yield only one retinal molecule. Despite its indispensable role in vision, it may furthermore play a role as a bioactive compound, due to its potential antioxidant effects [25], and its interaction with nuclear receptors, mainly RAR/RXR, which is important for cell differentiation and immunity [26]. These properties make β-carotene one of the most investigated biological molecules, both in academia and industry. Though its multifunctionality in humans is yet to be fully understood, several epidemiologic studies have demonstrated its relationship to a decreased incidence of chronic diseases such as blindness [27], xerophthalmia [28], cancer [29], cardiovascular diseases [30], diabetes [31] and premature death [32] and found to have an antioxidant component.

### 1.3. Challenges Associated with β-Carotene Food Fortification

β-Carotene is naturally found in various foods and is also commonly used as a natural pigment in food, pharmaceutical and cosmetic industries. This lipophilic molecule is characterized by the presence of a polyene structure with 11 conjugated double bonds with two β-ionone rings. Under environmental stress (temperature, humidity, pH, ionic strength and radiation), β-carotene may undergo transformation, resulting in the formation of different isomers such as 15-*cis*-β-carotene, 13-*cis-*β-carotene and 9-*cis*-β-carotene and several *trans*-β-carotenes [33,34]. *Cis*-isomers have bent structures and are likely to be more readily solubilized and adsorbed compared to *trans-*β-carotene which possesses a linear and rigid structure and has a high tendency to crystallize and aggregate as compare to the *cis*-isomers [35,36]. The unsaturated structure makes β-carotene prone to oxidation, resulting in the loss of its vitamin A functionality. Furthermore, β-carotene is also susceptible to isomerization when confronted with acidic conditions, high-salt, temperature, metal ions, peroxides and radiation during food processing and storage before consumption [36]. In addition, naturally occurring β-carotene is often complexed with protein molecules which limit its solubility and distribution in the food matrix, as well as its adsorption in human body [37].

Currently, β-carotene is one of the most exploited carotenoids and is usedto develop functional foods [38], formulate pharmaceutical supplements and prepare cosmetic products. However, food fortification, i.e., incorporating β-carotene within functional foods, is recognized as the most natural, appropriate and safe methods as compared to other drug administration routes including intravenous, intramuscular and subcutaneous ones [39]. However, within these functional food products, β-carotene is prone to physicochemical degradation during the production, processing and storage before food consumption. These limiting factors, in addition to its low bioavailability within the human gastrointestinal tract, make β-carotene difficult to incorporate into the food matrix and hence significantly impact its efficacy as a health beneficial plant compound.

Nanotechnology seems to be a logical solution to address these limiting factors, as it has demonstrated its potential to encapsulate, protect and delivery bioactive compounds using several delivery systems to improve their physicochemical stability, solubility, dispersibility and bioavailability upon ingestion [40,41,42,43,44]. Researchers have nanoengineered various kinds of delivery systems, such as microemulsion, liposomes, solid lipid carriers, nanostructured lipid carriers, nanocapsules and nanospheres to encapsulate and deliver bioactive compounds. These delivery systems are capable of improving stability, dispersity and bioavailability of bioactive compounds within the target food matrix. Although several excellent reports have already been published emphasizing the factors affecting the chemical stability of carotenoids [45], encapsulation techniques to protect them against environmental stress [46], production methods to prepare nanoengineered delivery systems [47] and delivery systems to improve their solubility or bioavailability [48], there is lack of reviews regarding β-carotene delivery systems, in particular with food applications.

The present article aims to contribute to the state of knowledge on the delivery systems used for β-carotene to improve its stability, solubility, dispersibility, bioavailability, as well as the development of functional foods. Before opting for designing an oral delivery system for β-carotene, it is paramount to understand its metabolism (digestion and absorption) as well as the factors affecting the physicochemical attributes of delivery system and their health risk and safety issues. Additionally, this review article will lead to a better understanding of the evolution of delivery systems for the encapsulation of β-carotene in food science.

## 2. Methodology

To search the literature, three most popular search engines of food and medical sciences, Google Scholar, Science Direct and PubMed as well as Scopus database were employed with the keywords “β-carotene”, “β-carotene encapsulation”, “β-carotene delivery system”, “engineered nanomaterial and β-carotene”, “β-carotene bioavailability”, “oxidative stress and β-carotene”. The timeline search (year) was: (a) 1900–1990, (b) 1991–2000, (c) 2001–2010 and (d) 2011–2020 in these search engines. After searching each keyword in the mentioned timeline, the first 100 most relevant entries were screened with direct observation. Adopting this method of literature search, nearly 2400 articles were screened and, based on the relevance of the topic, nearly 400 articles were summarized in the present review. The articles on food applications were prioritized in this review.

## 3. β-Carotene Metabolism

The fate of β-carotene in the human gastrointestinal tract (GIT) is determined by various factors, including the complexity of the ingested food matrix, its release from the food matrix, the transfer of the released molecule to the oil phase, its incorporation into mixed micelles, the entrance route into enterocytes and its incorporation into chylomicrons [49]. In the following, these processes are briefly explained.

### 3.1. Release of β-Carotene from the Food Matrix

Release of β-carotene from the food matrix is a multistage process, which begins by mastication in the mouth, followed by enzymatic and physiochemical process in the stomach and the small intestine [49]. The release of β-carotene begins with the physical disruption of ingested food particles in the buccal cavity of GIT to make β-carotene bioaccessible for absorption.

Bioaccessibility is defined as the quantity or extent of β-carotene that is released from food matrices in the gastrointestinal tract and remains bioavailable for absorption in intestine;

Bioaccessibility = Br×100Bt−Be

where, Br represents quantity of β-carotene released in GIT fluid in consequence to food matrix digestion, $B_{t}$: total quantity of β-carotene existing in the food matrix, and ${\;B}_{e}$: β-carotene secreted in the duodenal compartment along with bile salt.

The complexity of the food matrix has a great impact on the bioaccessibility as well as bioavailability of β-carotene, as its release from food the matrix is the major limiting factor for its bioavailability [37,49,50,51,52].

**Figure 1:**
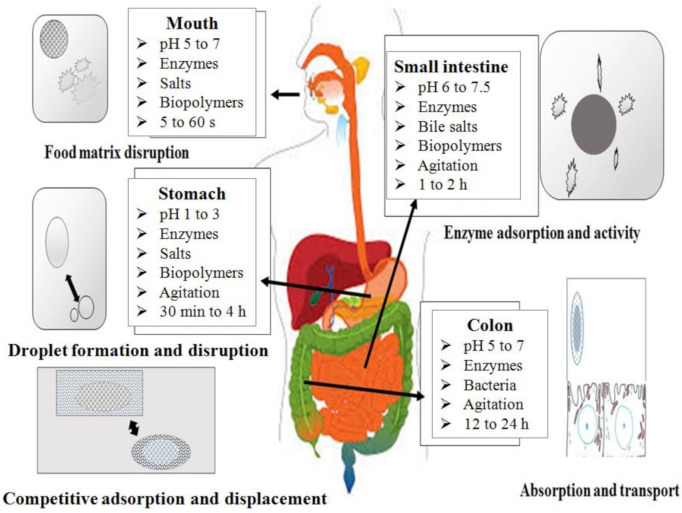
Schematic diagram of the human digestive system and the various physiochemical and physiological processes involved in the digestion and absorption of β-carotene.

The bioavailability of lipophilic compounds such as β-carotene can be defined as the part of the ingested β-carotene that is eventually recovered in the systemic (blood) circulation as an active form. Only then will β-carotene be available to travel to the target tissues and organs where it can exert beneficial health effects. For ingested β-carotene, there are several limitations that limits the amount that is distributed in the systemic circulation in its native form—e.g., chemical instability during the digestion process, poor solubility in the gastrointestinal tract (GIT), slow uptake from the GIT, cleavage by BCO1 in the enterocyte (producing 2 retinal molecules) [53], and first-pass metabolism (Figure 1). The oral bioavailability (F) of encapsulated β-carotene in delivery systems can be determined by the following equation:

$$F=F_{B}\times {\;F}_{A}\;\times {\;F}_{M}$$

where, F_B_ is the fraction of consumed β-carotene that survived passage through the upper GIT and is released from the food matrix/delivery system into the GIT, thus becoming bioaccessible for uptake by brush-bordered enterocytes. F_A_ is the fraction of the bioaccessible β-carotenethatis eventually absorbed by the enterocytes and then reaches the portal blood or, rather the lymph (and thus the systemic circulation). F_M_ is the proportion of absorbed β-carotene, which is preserved in its active form after first-pass metabolism in the GIT and the liver (and any other forms of metabolism or breakdown).

Naturally, β-carotene is present in different physical forms within chloroplasts and chromoplasts. In the chromoplasts, β-carotene is available either in crystalline form (e.g., in carrots and tomatoes) or in oil droplets (mango and papaya). It was noticed that bioaccessibility of β-carotene dissolved in oil droplets (10.1% for mango and 5.3% for papaya) was higher as compared to the crystalline form (3.1% for tomato and 0.5% carrot) [54].

The release depends on the degree of structural disruption of the food matrix, which can be enhanced by subjecting various food processing techniques (mechanical and thermal) before ingestion. It is believed that mechanical processing (homogenization, cutting, crushing and pureeing) may significantly improve bioavailability as it reduces food particle size, hence offering a greater surface to volume ratio for digestive fluids and enzymes to act upon, resulting in a higher release of β-carotene [49]. An 18% higher bioavailability (in vitro) in homogenized carrot as compared to chopped raw carrot supports this assumption [55]. Similarly, a two-fold higher bioaccessibility (in vitro) was witnessed for a 125 nm particle size as compared to a particle size of 126–160 μm [55].

Thermal treatments are also considered to be a good option for improving bioavailability, as they facilitate softening and disintegration of plant tissues and denaturation of β-carotene–protein complexes. Rock and team observed 3-fold increases in β-carotene serum levels when spinach was incubated for 40 min at 120 °C after canning and sterilization [56]. Similarly, commercially available carrot puree (subjected retort processing after cooking) has shown a higher bioavailability (in vivo) as compared to the carrot puree meshed in a grinder after 40 min of boiling [57]. Additionally, carrot that was finely peeled and chopped after boiling at 100 °C for 15 min was found to be more effective in raising the β-carotene serum level as compared to raw carrot [58]. Differences between the bioaccessibility observations from in vitro and in vivo bioavailability studies, such as higher bioavailability found in vivo, may be attributed to differences in food preparation methods and gastrointestinal simulation methods chosen, plus the inherent limitations of all in vitro methods [36].

Comparing various treatments, the thermal treatments were found to be often more effective in improving the bioavailability of β-carotene versus mechanical processing [59]. It was also assumed that the simultaneous application of thermal and mechanical processing may offer better release of β-carotene from food matrix. This assumption was supported when researchers observed a higher increase in β-carotene serum levels when fed with food subjected to homogenization and thermal treatment as compared to thermal processed or mechanical process food alone [60]. From the above observation, it can be postulated that the bioavailability of β-carotene is a function of particle size as well as of thermal processing. The improved bioavailability of β-carotene after simultaneous application of thermal and mechanical processes could be attributed to a reduction in particle size due to homogenization and degradation of β-carotene–protein complexes by thermal processing [37,60].

### 3.2. Mass Transfer to Oil Phase

Once β-carotene is released from the food matrix, it is either solubilized into oil phase or forms emulsion before the absorption. Several factors drive the transfer of released β-carotene into the oil phase [50,61]. The availability of the oil phase in the digesta is the primary limiting factor for the mass transfer of β-carotene into the oil phase, which may not accessible due to incomplete digestion of ingested food in the stomach resulting in incomplete release of oil phase [62]. Reduced particle size also improves its transfer, as it offers a greater surface to volume ratio, hence facilitating the partition of released β-carotene into the oil phase of the digesta [50,63]. In contrast, soluble proteins may limit the bioavailability of β-carotene as they hinder the incorporation of β-carotene into emulsions resulting after gastric digestion. Addition of 30% and 60% raw supernatants, containing soluble proteins, to blanched carrot juice resulted in 10% and 20% reductions in β-carotene transfer to the oil phase [63]. Further, it was also observed that the decrease in surface charge on emulsions (by reducing pH) improved the solubilization of β-carotene in the oil phase. Moreover, it is believed that low pH reduces the solubility of soluble proteins, resulting in acceleration in the rate of transfer of β-carotene to the oil phase. Rich and team recorded a one-hour increased transfer to oil in case of in vitro digested digesta at pH 2.1 as compared to in vitro digested digesta at pH 6.2 [64]. However, it has also been reported that under some conditions, proteins can aid in the emulsification of carotenoids including β-carotene in the digesta, improving its transfer into lipid droplet and thus later intestinal bioaccessibility [53]. This seemed to be the case especially under marginal digestion conditions—i.e., under low enzymatic digestive activity. It appears that both positive (emulsifying) and negative effects (by hampering, e.g., enzymatic access to protein-coated lipid droplets) are present, and depend on individual digestive conditions, testmeals, and carotenoids, whose effects overwhelm others [65].

In addition, the solubility of β-carotene in the oil phase, the amount of β-carotene in the digesta, quantity of oil ingested and foodmatrix aspects equally determine the amount and rate of transfer of β-carotene to the oil phase [64]. For example, dietary fiber is alleged to be a vital factor limiting the transfer of released β-carotene as it causes interference which: (i) hinders micelle formation; (ii) affects triacylglycerol lipolysis and emulsification of fat-soluble food compounds which facilitate the transfer of released β-carotene; (iii) limits the release of lipophilic nutrients from the fat droplets (oil phase); (iv) raises the viscosity of chyme, restraining the diffusion of lipophilic β-carotene from micelles into enterocytes [62,66].

### 3.3. Micelle Generation

The passage of the digesta into the small intestine stimulates the secretion of bile salts [50,67]. These bile salts (cholic, chenodeoxycholic, deoxycholic and lithocholic acids) have high surface activity, which aids in converting small lipid droplets into mixed micelles. The surface-active nature of these bile salts further improves the incorporation of β-carotene into mixed micelles by reducing their sizes to about 80 Å [68]. The incorporation of β-carotene into mixed micelles is regarded as obligatory for its uptake by the intestinal epithelium, as it ensures aqueous solubility and the diffusion to the unstirred water layer. Hence, factors affecting mixed micelle formation can significantly impact the bioavailability of β-carotene. An array of factors affecting the formation of micelles has been reported, including the amount of lipids in the digesta [56,69,70], type of fatty acids [71], degree of unsaturation and length of fatty acid [71], presence/absence of dietary fibers [49], and the presence of high amounts of minerals [72,73].

Dietary fat is one of the most important factors, as it not only facilitates the incorporation of β-carotene into mixed micelles, but also stimulates the secretion of bile salts. Prince and Frisoli [74] reported a 2.5-fold increase in β-carotene serum levels 40 h postprandial when β-carotene was ingested along dietary fat as compared to β-carotene ingested without dietary fat [74]. Furthermore, a rise in β-carotene serum levels (and other carotenoids) was also recorded when salad was ingested along with avocado oil (24 g) or avocado (150 g avocado) compared to salad alone [75]. A rise in β-carotene serum level of human subjects was also noticed when they were fed with β-carotene (8 mg) along with increasing quantity of hot bread spread (from 3 g to 36 g) [69]. In total, these results clearly indicate that there must be a minimum threshold for the amount of dietary fat present in test meals to enable optimal β-carotene absorption, an amount which is likely at least 3 g of dietary fat for the uptake of β-carotene for a typical meal containing approx. 8 mg of carotene. Nevertheless, the proposed threshold (3 g fat for 8 mg β-carotene) still remains a matter of debate and is likely to depend on matrix factors and perhaps host factors. Moreover, Castenmiller and his team proposed 5 g of fat per meal for optimal absorption of β-carotene [70]. This proposal was also supported by Hedren et al. [55] when adding 20% of cooked oil into homogenized carrot pulp improved β-carotene in vitro bioaccessibility by 27% [55]. In addition to the amount of dietary fat, the chain length of fatty acids equally influences micelle formation, as well as β-carotene incorporation within the mixed micelles. Hugo and team registered a significant increase (4.9 to 8.6 to 14.9%) in micelle efficiency with increased fatty acid chain length from butanoic acid (4) to octanoic acid (8) to oleic acid (18), respectively. This may not be surprising, given that short- and even medium-chain fatty acids can be absorbed via the portal vein [76], and do not necessarily contribute to mixed micelle formation. Moreover, the degree of unsaturation in fatty acids has also shown significant impact on bioavailability—e.g., a higher bioavailability of β-carotene was observed when it was ingested along with unsaturated vegetable oil when compared to saturated vegetable oil [77]. In contrast, the micelle efficiency was not significantly influenced with increase in degree of unsaturation from 1 (oleic acid, c18:1) to 3 (linoleic acid, c18:3) [77].

As for matrix release and oil droplet incorporation, dietary fiber is thought to limit β-carotene bioavailability. The inhibitory effect of dietary fibers on β-carotene bioavailability has been demonstrated by several in vivo and in vitro studies [67,78,79,80]. These could be attributed to a number of factors, including hindrance in micelle formation, alteration on triacylglycerol lipolysis and emulsification of lipophilic compounds, and finally, restraining the diffusion of β-carotene from mixed micelles to enterocytes.

### 3.4. Absorption

Following diffusion through the mucus layer in the small intestine, micelles incorporating β-carotene come into contact with enterocytes, eventually resulting in the uptake of β-carotene into the cytosol of the enterocyte. Absorption of β-carotene is thought to be a concentration dependent process—i.e., at lower concentrations it absorbs via protein transporters including cluster determinant 36 (CD 36) and scavenge receptor class B type 1 (SR-BI), while at higher concentrations it follows passive diffusion [81].

Passive diffusion is thought to be the primary mechanism for β-carotene absorption and is mediated by the difference between micelles and plasma membranes of enterocytes [49,50,81]. Viscosity is also thought to be a limiting factor for this diffusion process, as it interferes with the mobility of the mixed micelles [82]. Several other factors, such as physiochemical state of β-carotene (molecular forms, potency and their physiological linkages), presence of lipophilic compounds, phytosterols, soluble proteins, surface-active compounds (phosopholipids/surfactant), inhibitor/enhancer β-carotene and host-related factors (age, disease, surgery, obesity, genetic variation) are equally responsible for influencing the bioavailability of β-carotene, by a variety of factors such as competitive mechanisms, SNP expression, available surface for absorption etc., which have been comprehensively reviewed in our previous articles [49,83]. After absorption, β-carotene needs to be incorporated into chylomicrons before entering the lymphatic system and systemic circulation [37,61]. The transport through the cells has been the topic of some discussion but has not been fully elucidated. It may include unidentified transport proteins, BCO1, retinol binding proteins, and others [84,85,86].

## 4. Bioavailability Assessment

Determining the bioavailability in human subjects is considered to be ideal, but it seems to be impractical in many cases as the results of bioavailability studies may vary due to large variations among the population, cost issues, non-compliance of ethical restriction and time-consuming nature of experimentation. In vitro digestion models are gaining popularity as they are reproducible, rapid and allow handling of a large number of samples in parallel. Even though in vitro digestion protocols to evaluate the bioavailability of bioactive agents (including β-carotene) have been developed and advanced in the last decade, there are still some controversies around standard digestive models that can be used for assessing β-carotene bioavailability.

Selection of a suitable in vitro digestion model is the first stage for evaluation of the bioaccessibility of a nutrient. Currently, two types of in vitro digestion models—static and dynamic models—are primarily employed for determination of the bioavailability of bioactive compounds [49,51]. The static digestion models rely on a set of physicochemical conditions (pH, bile salt concentration, enzyme) occurring during the digestion process without imitating peristalsis, fluid flow and thorough mixing occurring during digestion. Dynamic models rely on mechanical forces that occur during digestion along with imitation of the enzymatic and chemical changes (changes of enzyme, mineral and bile concentrations and pH) over time and between the different compartments. Dynamic models offer better control over pH, enzyme concentration and mechanical forces, but are more difficult to set up. Selection of suitable digestion models solely relies on the scope of measurements as well as the nature of samples to digest. Discrepancies in the measurement of β-carotene bioaccessibility between such methods have been reported—e.g., from almond butter by dynamic in vitro digestion (87.1%) versus a static model (51.0%) [87]. These observations suggested that static in vitro models suit simpler samples with perhaps higher throughput, while dynamic in vitro digestion models are more suitable for solid or semisolid food matrices. Several in vitro models (gastric as well intestinal) have been applied to determine β-carotene bioavailability, which were primarily derived from the model proposed by Garret [88]. Each model has its advantages and limitations, which have been comprehensively reviewed in our previous article [49]. However, a huge step forward was made with the proposed INFOGEST consensus model, published in 2014 [89] with a follow-up update a few years later [90], which was based on both physiological meaningful conditions as well as practicability aspects.

Several factors, including food composition, complexity of the matrix, degree of processing and genetic variations play vital roles in the bioavailability of β-carotene [36,83]. Generally, when β-carotene is released from food matrix, it has to be incorporated into oil droplets, either formed during lipid digestion or present in the original food (e.g., emulsions). The attachment of lipases from digestive juices at the oil droplet surface initiates lipid digestion. The digested lipid products, particularly some free fatty acids and monoacylglycerols, take part in the formation of mixed micelles (also containing bile salts and phospholipids), which behave as carriers to solubilize β-carotene and transport it to the epithelium cells before adsorption [61]. Therefore, the ingestion and hydrolysis of lipids have been regarded as essential steps in the bioavailability of β-carotene [91,92]. Technically, any factor that influences lipid digestion would affect the bioavailability of β-carotene.

### Improving Bioavailability of β-Carotene by Encapsulation

**Figure 2:**
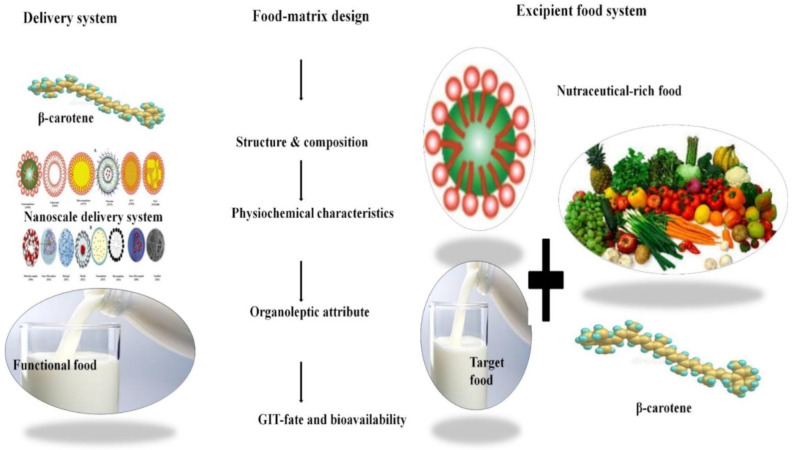
Strategy to improve the bioavailability of lipophilic constituents in foods.

A variety of foods are being fortified with β-carotene. Direct addition of β-carotene in food may result in inescapable interactions that lead to compromises regarding food quality, taste, appearance and the bioavailability of β-carotene that can significantly diminish its efficacy as a disease-combating agent [93,94]. In addition, the obligatory role in human health and the mentioned physico-chemical challenges of β-carotene drive the development toward more efficient, biocompatible, and safer delivery systems, with greater patient compliance, such as using nanotechnology for better incorporation in target foods (Figure 2) [95]. These challenges open new windows of opportunity to food technologists to utilize nanotechnology and to develop β-carotene delivery systems that do not compromise food quality. Encapsulation is regarded as an indispensable process to fabricate delivery systems with improved bioavailability, by stabilizing β-carotene in the target foods and also during gastrointestinal (GIT) passage, improving its solubility in digestive fluids, hence enhancing its absorption from the GIT, and possibly even evading first-pass metabolism loss in various tissues. The bioavailability of encapsulated lipophilic compounds including β-carotene is compromised by a range of factors and has been reviewed by various researchers in excellent reviews [46,48,51,55,96,97,98,99].

In order to attain the desired solubility, dispersity, stability and bioavailability for β-carotene, a range of delivery systems, differing in design, structure, composition and production processes, have been tested to validate their potential to encapsulate β-carotene and to be an efficient carrier for β-carotene delivery in food systems [51]. From the origins of nanostructures such as delivery systems for β-carotene to the present date, the number of publications based on delivery systems has significantly increased. There are three major reasons that can explain their success: (i) the improvements in delivery system development; (ii) advancements regarding innovative technologies for delivery system synthesis avoiding organic solvents; (iii) applications of newly developed drug delivery systems for food applications.

The success of the inclusion of delivery systems encapsulating lipophilic compounds such as β-carotene in food items solely relies on the following targets [100,101]: (i) reduction in solubility complications between β-carotene and the food matrix; (ii) protecting β-carotene against pH, temperature, moisture, oxidation and other detrimental external environment conditions; (iii) demonstrating improved bioavailability, also considering the potential for controlled and site-specific release of encapsulated β-carotene; (iv) avoidance of interferences with desired physiochemical properties of the food system.

## 5. Delivery Systems for β-Carotene

β-Carotene is often used as a natural colorant and additive in food in spite of having poor water solubility, a high melting point, susceptibility to environmental conditions, chemical instability, heterogenous distribution in food matrices, and low bioavailability—all factors that limit its potential for the food industry. In this regard, encapsulation techniques have allowed researchers to develop a range of delivery systems with desired functionalities, such as enhanced stability, high dispersibility, improved solubility and targeted/controlled release and improved bioavailability [102,103].

Delivery system is the technology where a bioactive ingredient is enclosed in nano-/microstructure not only to protect bioactive compounds against environmental degradation (oxidation, pH and enzyme), but also to release them at a particular target site in a defined rate [51]. At present, the most investigated delivery systems adopted for β-carotene can primarily be categorized into two groups: polymer-based delivery systems (PBDSs) and lipid-based delivery systems (LBDSs).

### 5.1. Polymer-Based Delivery Systems

**Figure 3:**
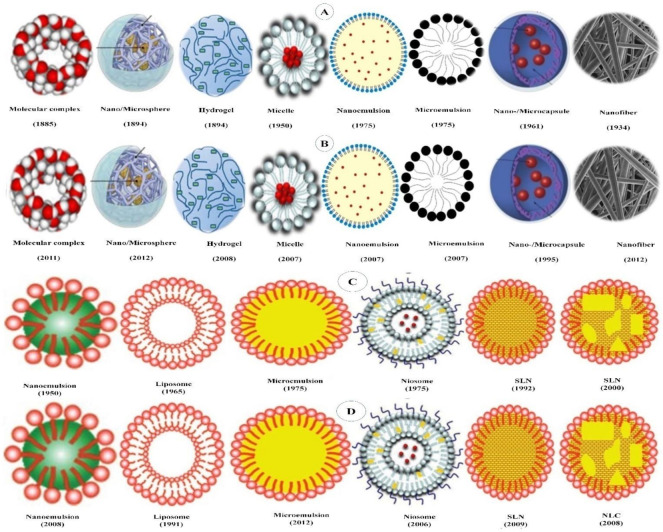
(**A**) Historical event in the evolution of polymer-based delivery systems; (**B**) historical event in the application of polymer-based delivery system for encapsulating β-carotene; (**C**) historical event in the evolution of lipid-based delivery systems; (**D**) historical event for applying lipid-based delivery system for encapsulating β-carotene.

Polymer-based delivery systems use the intrinsic diversity of polymers to develop encapsulating bioactive compounds in nanodelivery with improved functionalities. The long-term health risks of PBDSs either fabricated with a synthetic polymer or made up of natural polymers, such as proteins and carbohydrates, are regarded as minimal. However, the latter are either hard to scale-up as they require several heat and often complex treatments which are hard to control or result in porous micro-/nanoparticles, thereby not achieving the objective of encapsulation. A range of PBDSs have been reported in the literature. In the present review, we have included only those PBDSs which are derived from either natural food grade materials or are generally recognized as safe polymers. Typical PBDSs include nano-/microspheres, nano-/microcapsules, hydrogel micelles, colloidal nano-/microemulsions and nanofibers, all of which mainly consist of synthetic or natural polymers (Figure 3A,B).

#### 5.1.1. Inclusion Complexes

Inclusion complexes are one of the most adopted delivery systems for encapsulating bioactive compounds. The complex formation between the bioactive compound and the host molecule occurs only in the presence of water. Cyclodextrin molecules are the most widely used host molecules for the preparation of molecular complexes. Cyclodextrins are macrocyclic oligosaccharides comprised of α(1,4)-linked glucopyranose subunits that contain a distinctive hydrophilic outer surface and a lipophilic central cavity [104]. This molecule offers a cage-like supramolecular structure, which can interact with the structures of various lipophilic bioactive agents. Utilizing their ability to form inclusion complexes with a range of “guest” molecules, cyclodextrins are recognized as being among the most important supramolecular host molecules [105].

The literature describes various methodologies such as solvent evaporation, chemical modification and isoelectric precipitation-fabricated inclusion complexes [106,107,108,109,110,111]. This paper focusses on those methodologies which allow the formation of β-carotene inclusion complexes (Table 1). Both human and animal studies suggest that cyclodextrins can be used to enhance lipophilic bioactive compounds such as β-carotene in food matrices [104,112,113,114]. There is only a single report on β-carotene encapsulation in cyclodextrins which was published in 2011 and assessed the solubility of cyclodextrincomplexes encapsulating β-carotene [113]. Furthermore, researchers have also utilized maltodextrin’s ability to encapsulate β-carotene [115]. Moreover, a research team also validated the suitability of the amylose molecules to encapsulate lipophilic β-carotene [116]. For this purpose, they encapsulated β-carotene in spherical microparticles (mean diameter—8 mm) using an emulsion method and carried out stability studies against oxidative stress (FeCl_3_), photodegradation and release kinetics in simulated digestive fluid (gastric as well as intestinal fluid) [116]. These amylose microparticles were not only able to retain β-carotene activity upto 70% as compared to nonencapsulated β-carotene after 7 h of UV exposure but also had higher stability (75% retention) as compared to nonencapsulated β-carotene (18%) after 7 h of FeCl_3_ exposure [116]. Further, simulated digestion studies also suggested that amylose microparticles were resistant to acid conditions (resistant to gastric digestion) but demonstrated high release (25% of encapsulated β-carotene) in simulated intestinal fluid during 3 h treatment [116].

Despite the high stability of entrapped bioactive compounds, molecular inclusion has several limitations, including poor release of the encapsulated bioactive compound, low loading capacity, as well as high cost and failure of legislative compliance, as cyclodextrins are not legally permitted in food systems in some countries. To deal with regulatory compliance, researchers have come up with specific carbohydrate molecules (amylose and maltodextrin) which display unique binding properties to lodged lipophilic ligands in their hydrophobic patches. These molecules (amylose and maltodextrin) offer high encapsulation and protection against oxidative, and chemical and photodegradation for β-carotene could be attributed to a three-way interaction: (i) the helical cavity/hydrophobic patches of these carbohydrate molecules demonstrate greater affinity for lipophilic β-carotene possibly due to their “slim” and hydrophobic alkyl chains and (ii) altered microparticles matrices’ viscosity profiles resulting in the formation of a soluble high molecular weight nanocomplex, and they (iii) offer better linkage for carbohydrate-surfactant-encapsulant compounds (β-carotene) in ternary structures [251].

#### 5.1.2. Micro-/Nanospheres

Micro-/nanospheresarederived from natural or synthetic polymers having particles size between 1–1000 µm (microspheres) and or 1–1000 nm (nanospheres). These are water-soluble polymer or mixture of polymers dispersed in an organic phase to form spherical structures in the presence of cross-linking agents. Bioactive compounds can be encompassed into the inner hollow core of nanospheres or entrapped in the polymeric matrix of a solid micro-/nanosphere.

Several methodologies for the preparation of nano-/microspheres, such as single emulsion, double emulsion, coacervation phase separation, and polymerization have been adopted for encapsulating various bioactive compounds [180,188,189,190]. These delivery systems are renowned for their ease of optimization to obtain the desired functionalities for pharmaceutical needs, including targeted and temporal control of release of encapsulated drug, efficacy and in vivo stability as well as biocompatibility.

In spite of the great potential in the pharmaceutical field for drug delivery, nano-/microspheres remain underutilized for β-carotene encapsulation. In order to obtain better knowledge about the role of nano-/microsphere for β-carotene delivery, we have discoursed about those methodologies that are involved in β-carotene encapsulation. The encapsulation of β-carotene in micro-/nanosphere was first carried out with a carrageenan/carboxymethyl cellulose-based microsphere to determine the release kinetics of encapsulated β-carotene from genipin-cross-linked kappa-carrageenan/carboxymethyl cellulose [153]. During course of time, several studies were carried out to evaluate the potential of polymeric micro-/nanospheresas an alternative delivery system for β-carotene encapsulation [188,191,192,193,194,195,196,197,252]. Nevertheless, there is a scarcity of data on the use of nano-/microsphere for the purpose of β-carotene fortification in food systems. Though, these micro-/nanospheres are relatively easy to scale-up as they do not require sophisticated instrumentation. However, several challenges such as poor loading capacity [253], premature release and degradation by enzymes [254] could be the reason for micro-/nanospheres not being among the more accepted species for the encapsulation of β-carotene.

#### 5.1.3. Nanohydrogels

Nanohydrogels, three-dimensional soft gels, are generally made by cross-linking the water-soluble material, which is comprised of a wide range of chemical compounds and bulk physical properties. The use of hydrogels as a delivery system results in a number of advantages, including reduced systemic side effects [255], sustained and site-specific drug delivery under desired external stimuli (thermal, pH or mechanical changes) [256] and reduced systemic side effects attributed to loss in encapsulated bioactive compounds (β-carotene) during digestion and inevitable interaction with other components of food matrices, hence offering improved bioavailability [257]. The literature has been updated with excellent reviews on preparation methods for nanohydrogels including sonication methods, cross-linking and inverse-suspension polymerization [258,259,260].

Chu et al. [159] compared the suitability of sodium caseinate- (SC) (mean diameter 17 nm) and whey protein-based (mean diameter 45–127 nm) hydrogels to protect encapsulated β-carotene against physicochemical stress including heat, salt and pH [159]. It was observed that β-carotene encapsulated within sodium caseinate-based hydrogels had higher stability (minimal change in particle size and zeta potential) as compared to whey protein-based hydrogels against various stress conditions [159]. Similarly, β-carotene-loaded κ-carrageenan hydrogel was also synthesized and tested for photodegradation, thermal stability and simulated digestive release kinetics. It was observed that approximately 75% of encapsulated β-carotene was retained in κ-carrageenan hydrogel after 24 h of UV exposure, while approximately 89% of encapsulated β-carotene was found to be retained when they were incubated at 4 °C as compared to hydrogel incubated at 25 °C (>35%) [261]. Further, alginate nanohydrogel was found to be more effective in providing stability to β-carotene under accelerated storage conditions (55 °C), bioaccessibility and bioavailability as compared to β-carotene encapsulated in nanoemulsion [243]. The high structural and chemical stability of the developed hydrogel system against pH, heat and salt, encouraged further progress in designing hydrogels as an efficient delivery system for β-carotene (Table 1) [115,153,243,244,245,246,247,248,262]. Nevertheless, the great potential hydrogel also carries several limitations including poor loading capacity [257], premature release and oxidation of β-carotene [153,223]. These could be among the reasons that hydrogels have not been well adopted as species for the encapsulation of β-carotene for food applications.

#### 5.1.4. Micro-/Nanocapsules

Micro-/nanocapsules belong to the vesicular system family in which the bioactive compound is situated within a cavity comprised of an inner liquid core fenced by a polymeric membrane, with a range of sizes, microspheres (1–1000 µm) and nanospheres (1–1000 nm). Solvent displacement and spray-drying are some of the well adopted techniques for fabricating nano-/microcapsules. These delivery systems are recognized as substitutes to liposomes due to its cost-effective and triggered release under specific stimuli.

The first report on the use of microcapsules to encapsulate carrot-derived β-carotene was published on β-carotene-loaded microcapsules which were prepared by using spray-drying to evaluate the effectiveness of microcapsules to retain encapsulated β-carotene [223]. In the following, a research team developed β-carotene-loaded nanocapsules (different in gum Arabic concentration 15 to 30%) to study the impact of the effect of increased gum Arabic concentration (15 to 30%) on the stability of β-carotene and it was found that microcapsules fabricated with 25% gum Arabic had highest retention capacity for β-carotene [224]. Thereafter, various reports have been published on the production of micro-/nanocapsules [154,225,226,227,239,263,264] (Table 1).

Despite these gained insights, only few food technologists have prepared β-carotene-loaded nanocapsules that are suitable for the purpose of food applications [150,151,152,153,154,225,226,227,239]. This could be because of their operative limitations such as complexity in their fabrication process [265], the use of synthetic polymers [266] and the susceptibility for leakage of β-carotene which is adsorbed on their surface or can be imbibed within the polymeric membrane [267]. These limitations are also further aggravated by the failure of technology to resolve stability issues such as aggregation, fusion, leakage and sedimentation. Once these aforementioned limitations are addressed and solved, there is great potential for micro-/nanocapsules to act as efficient delivery systems for β-carotene in food applications.

#### 5.1.5. Nanofibers

The exclusive properties of nanofibers such as their nanoscale dimensions, quick wetting properties, rapid release and temperature independence nature makes nanofiber-based delivery systems a good technique for the delivery of heat sensitive bioactive agents such as β-carotene [268]. Electrospinning, freeze-drying and centrifugal spinning are extensively adopted encapsulation techniques for heat susceptible bioactive compounds [269,270]. A range of wall materials are used to fabricate nanofibers broadly categorized into two classes—(i) natural and (ii) synthetic. Natural wall materials involve cellulose, chitosan, pullulan, cyclodextrins, starch, gelatin, zein protein, egg albumin, soy protein, and whey protein while synthetic wall materials include polyvinyl alcohol, cellulose acetate, hydroxypropyl methyl cellulose, ethyl cellulose, methyl cellulose [271,272].

Despite these promising properties, nanofibers have remained untapped for encapsulating β-carotene. It is evident that there is a scarcity of reports addressing β-carotene encapsulation in nanofibers [117,198,241,242,273]. One major reason is that the porous nature of nanofibers makes them liable to oxidative degradation of β-carotene, which makes it unfit as a delivery system for β-carotene encapsulation [271].

### 5.2. Lipid-Based Delivery Systems

Lipid-based delivery systems (LBDSs) involve delivery systems which are principally composed of physiological lipid analogs such as surfactants as stabilizers (Figure 3A,B). LBDSs have been recognized for their promising biocompatibility, competency in GIT penetration, easy to scale-up and broad application [102,274]. LBDSs have been admired for their potential for drug delivery through various administration routes, particularly for the oral delivery of lipophilic drugs, because of their competence to mimic the food lipids during the digestive process [275,276]. With their properties, lipid-based delivery systems offer an array of advantages over polymer-based systems as shown in Table 2. Some of these advantages of lipid-based nanodelivery systems entail: (i) biocompatibility and use of nontoxic excipients [274,277]; (ii) high drug payload [143]; (iii) viability of incorporating both lipophilic and hydrophilic bioactives [274]; (iv) prospect of controlled release and drug targeting; (v) improved drug stability [278]; (vi) averting of organic solvents [279]; (vii) cost-effectiveness [280]; (viii) ease of scale-up during production and sterilization [95]. Over the course of time, a range of lipid-based delivery systems have been developed for encapsulating bioactive compounds such as micelles, micro- and nanoemulsions, liposomes, niosomes, solid lipid carriers, nanostructured lipid carriers, bilosomes, cubosomes, etc. [281]. However, in the present review, the emphasis has given those LBDSs which have been adopted for encapsulation β-carotene are discussed in the following sections.

#### 5.2.1. Micelles

Micelles are distinguished as colloidal dispersions (with particle sizes ranging between 5 to 100 nm), related to a large family of dispersed systems containing particulate matter (called the dispersed phase), distributed within a continuous phase [282]. The hydrophobic regions of amphiphilic molecules form the core of the micelle while hydrophilic regions form the micelle’s shell. When micelles are used as delivery systems for lipophilic β-carotene in aqueous phases (food items and beverages), fat-soluble molecules are imbibed on the micelle surface [283].

Several researchers have reproduced excellent reviews highlighting the chronological developments in the design, preparation, characterization and evaluation of polymeric micelles to attain efficient delivery of lipophilic drugs [284,285,286,287]. Micelles promise an array of advantages over polymeric nanoparticles, such as higher water solubility to lipophilic bioactive compounds [288], better penetration across physiological barriers [289], reduced toxicity and other adverse effects and effective bioactive drug distribution among tissues as well as organs [47,290]. These attractive attributes fascinated food technologists to exploit β-carotene encapsulation. Chu et al. [174] encapsulated β-carotene in sodium caseinate-based micelles to correlate the changes in the particle size and ζ-potential of the nano dispersions with their composition [174]. These β-carotene-loaded micelles displayed a better stability than that of empty micelles [174]. β-Carotene-loaded α-lactalbumin micelles was not only found to be effective in protection of β-carotene (40% to total encapsulated β-carotene) against thermal degradation (after 24 h of incubation at 60°C) but also demonstrated high cellular uptake of micelles encapsulating fluorescent dye by Caco-2 cell which also signifies higher absorption of encapsulated β-carotene [291]. These observations attracted food technologists to encapsulate β-carotene in micelles, using different food grade ingredients including casein, α-lactalbumin, and β-lactoglobulin [160,175,176,177,178]. Low loading capacity, premature release of drugs and poor stability has nevertheless limited the use of micelles in food applications [47].

#### 5.2.2. Micro/Nanoemulsions

Oil-in-water nanoemulsions and microemulsions are two basic colloidal dispersion systems suitable for the delivery of lipophilic β-carotene for food applications. The literature also reports several techniques for the preparation of micro/nanoemulsions, such as emulsion phase inversion [292], high-pressure homogenization [293], microfluidization [144,294], supercritical fluid methods [145,295], spontaneous emulsification [296] and phase-inversion temperature [297].

Micro/nanoemulsions are recognized as colloidal dispersion systems of small liquid droplets, depending on the size (≤100 nm for microemulsion and ≤50 nm for nanoemulsion) [298]. The main difference between these two kinds of colloidal systems is thus their thermodynamic stability—i.e., microemulsion being thermodynamically stable while nanoemulsion being thermodynamically unstable [298]. It is assumed that the type of carrier oil and degree of saturation have significant impact on the β-carotene bioaccessibility. For this purpose, β-carotene was encapsulated in three different nanoemulsion differing in their carrier oil (long-chain fatty acid, medium-chain fatty acid and orange oil) and it was found that nanoemulsion derived from long-chain fatty acid had higher bioaccessibility (≈66%) as compared to medium-chain fatty acid (≈2%) and orange oil (negligible) [92]. Teapolyphenols (TPs) nanoemulsion was also fabricated to encapsulate β-carotene with the hypothesis that being an antioxidant itself the TP could protect the encapsulated β-carotene. It was observed that addition of TP prevented the degradation of β-carotene during storage and improved the bioaccessibility of β-carotene after simulated oral and stomach digestion [299]. These observations have encouraged food technologies to develop noval nanoemulsions incorporating β-carotene [91,125,140,141,142,143,144,145,146,147,149]. Nevertheless, β-carotene incorporation into nanoemulsions and microemulsions for food applications has shown to be limited due to technical and practical hurdles, such as scarcity of food grade surfactants [300], complexity in fabrication method (most of them involving organic solvents), poor loading capacity and instability during storage [301].

#### 5.2.3. Liposomes

In general, liposomes are spherical liquid structures with an aqueous core enveloped by as single (unilamellar) or multiple lipid bilayers (multilamellar liposomes) and promise high biocompatibility with animal tissues as they have demonstrated similarity to natural plasma membranes. According to the size, they are also defined as nanoliposomes (≤200 nm). The ability to incorporate both hydrophilic and hydrophobic compounds individually or simultaneously make liposomes most adopted delivery systems. Their broad application is also endorsed by their structure flexibility, size and composition. Various fabrication methods for preparation of liposomes have been developed, including lipid film hydration, microemulsification, sonication, membrane extrusion, dried reconstituted vesicles, solvent dispersion method, detergent removal technique and supercritical fluid method [285,301,302,303,304,305,306,307,308,309].

Liposomes are one of most widely used delivery system to encapsulate and deliver lipophilic as well as hydrophilic bioactive compounds for cosmetics, pharmaceuticals and food industry [310,311]. It is assumed that the stability of encapsulated β-carotene can be further improved by the addition of antioxidants, though this may compromise the loading capacity. This assumption was varied for a study where β-carotene was found to be more stable (approximately 88%) when encapsulated along in liposome with vitamin C as compared to liposome without vitamin C (approximately 36%) during 30 days of storage at 4 °C [312].

Liposomes are comprised of a hydrophilic core and a lipophilic crust, thus being able to incorporate bioactive compounds differing in their hydrophilicity. Hence, the solubility of any bioactive compounds governs its loading capacity as well as its location within the liposome [118]. For instance, the loading capacity of β-carotene was compromised when β-carotene was encapsulated in liposomes along with additional antioxidants such as lutein and lycopene [313,314]. Xanthan gum-coated liposome has shown high retention ability for encapsulated β-carotene (2 molar β-carotene) during 90 days of storage under refrigerated conditions [119]. L-α-Dipalmitoylphosphatidylcholine-based liposomes was evaluated for release of β-carotene in simulated digestive system and it was observed that only 5% gum Arabic concentration 10% of total encapsulated β-carotene was released under gastric digestion conditions while 30–40% of total encapsulated β-carotene was released under intestinal digestive fluid [315]. Liposomes have also been reported improved stability for encapsulated β-carotene [117,118,119,120,121,122,123].

Though liposomes are the most widely adopted delivery systems for food bioactives it also has several limitations such as hard to scale-up due to their vulnerability to shear, sedimentation, aggregation, fusion and environmental stress (osmotic pressure, pH, temperature, oxidation, etc.), which may result in premature release and degradation of encapsulated β-carotene. To overcome this hurdle, food technologist came up with a proliposome strategy, nanometric version of liposomes, which offers more surface volume ratio, improved solubility to lipophilic compounds, enhance bioavailability, improve controlled release, enable site directed release of encapsulant, and high stability during processing and storage [316]. Regardless of their great stability, proliposomes also carry technical limitations, such as the need of a vacuum or nitrogen atmosphere during their fabrication and storage [317]. It is also evident that for these reasons the food industry has not adopted this technique. Additional challenges with liposomes/proliposomes include low water solubility, short half-life, sedimentation, aggregation, fusion and phospholipid hydrolysis and/or oxidation, and high production costs remain high [318].

#### 5.2.4. Niosomes

Niosomes are vesicles formed as a result of unfavorable interactions between nonionic surfactants and water molecules resulting in closed bilayer structures and can also encapsulate lipophilic, hydrophilic and amphiphilic compounds [319]. Niosomes are preferred over liposomes as they offer better mucosal permeability, sustained and site-specific release, higher stability and are cost-effective [320]. Niosomes promise higher chemical stability, simultaneous encapsulation of hydrophilic and hydrophobic bioactive compounds and reduced toxicity due to their nonionic nature [321]. They also resolve the issue coupled with liposomes such as challenges during sterilization, phospholipid purity and high costs [321]. In addition, the scale-up of nisomesare also simple, as they do not require any specific conditions, organic solvents and other precautions such as vacuum [284,309,322,323,324]. In spite of the great potential, niosomes are not well adopted for food fortification. There is a scarcity of data on niosome encapsulation of β-carotene and only a single study is available for β-carotene in niosomes where high stability for β-carotene was observed when it is encapsulated in noisome (20 µm) than that of dissolved in tetrahydrofuran (10 µm) after 96 h incubation at 50 °C [124]. Furthermore, it is evident that not a single report was generated on applications of niosomes regarding encapsulating β-carotene for the food industry. This could be due to failure in resolving major stability issues, such as aggregation, fusion, leakage and sedimentation that were also observed in liposomes.

#### 5.2.5. Solid Lipid Nanoparticles

Nanotechnologists have evolved next generation delivery system termed solid lipid nanoparticles (SLNs), where the liquid lipid (oil) has been substituted by a solid lipid [325]. SLNs promise exclusive properties such as a better interaction of phases at the interface, greater stability of encapsulated bioactive compounds, controlled and or/targeted drug release, large surface area and small size and ease in scaling up, which make it a promising delivery system for hydrophilic bioactives [326].

Studies are available on SLN fabrication methods, such as evaporation or diffusion [126,327], high-pressure homogenization at high or low temperatures (including cold homogenization and hot homogenization) [328], phase-inversion methods [328], solvent emulsification [329], supercritical fluid (supercritical fluid extraction of emulsions (SFEEs) [330], homogenization or high-speed assisted ultrasonication [331] and spray-drying [332].

The potential of SLNs to encapsulate and protect β-carotene was recognized during their initial development phase where β-carotene was incorporated into SLNs to evaluate the effect of surfactants on the oxidative stability of encapsulated β-carotene. It was also observed that high-melting surfactants better protected encapsulated β-carotene against chemical degradation [127]. It was assumed that the incorporation of protein molecules into SLNs also improves the stability of encapsulated β-carotene. In order to verify these aspects, a study was carried out to assess the impact of whey protein on the stability of encapsulated β-carotene in SLNs [128]. Though this study demonstrated better stability of β-carotene in SLN, there is scarcity of data on SLNs for food applications. Only one paper was published addressing β-carotene-loaded SLNs for food fortification [129]. Low drug loading capacity, drug expulsion after polymeric transition during storage, particle size growth, random gelation tendency, unforeseen dynamics of polymeric transitions and sometimes burst releases are some of the limitations of SLN [325,333].

#### 5.2.6. Nanostructured Lipid Carriers (NLCs)

NLCs contain an unstructured solid lipid core matrix, which consists of a mixture of liquid and solid lipids and an aqueous phase consisting of a surfactant or mixture of surfactants. Usually, liquid and solid lipids are a blend in a defined ratio that could vary from 70:30 to 99.9:0.1, while the surfactant content is kept between 1.5% and 5% (*w/v*) [334].

Current literature reports use various fabrication methods for NLCs including, high-pressure homogenization at high or low temperatures (including cold homogenization and hot homogenization) [335], solvent emulsification–diffusion techniques [331], supercritical fluids (supercritical fluid extraction of emulsion) [336], solvent emulsification evaporation [335], solvent displacement [135], solvent diffusion [337], phase inversion [338,339], melt emulsification [340], sonication [334], spray-drying [340,341], and solvent evaporation [335].

Among the aforementioned preparations methods, the hot homogenization process is preferred for NLC fabrications for food applications, as it does not involve organic solvents [342]. NLCs are partly crystallized lipid nanodelivery particles with an average diameter of ≤100 nm. The unstructured/partially solid matrix produces interesting nanostructures, which improves the stability of entrapped bioactive compounds, offers high loading capacity and controlled/target release [343]. It is believed that the addition of antioxidant aqueous and or lipid phase may increase the physiochemical stability of NLCs as well as the entrapped bioactive compound. Ethylenediaminetetraacetic acid and tocopherol were shown to offer better oxidative stability to carotenoids (astaxanthin), while ensuring higher physical stability of NLC particles [344]. It was also hypothesized that the surfactant and emulsifiers utilized in NLC preparations might negotiate the physiochemical stability of NLC particle as well as the encapsulated bioactive compounds. In order to verify this assumption, a study was devoted to formulating NLCs with two types of lipids differing in their melting points—i.e., low-melting (LM) and high-melting (HM) lecithins encapsulating tristerin and omega-3 fish oil [345]. The observation clearly suggests that NLCs formulated with HM lecithin demonstrated greater inhibition ability against oxidation of omega-3 fatty acids than that of LM lecithin [345].

Despite being a promising technique for drug delivery, NLCs remained underutilized for β-carotene encapsulation. To date, only a few dedicated reports have been produced dealing with β-carotene encapsulation in NLCs, showing the potential of NLCs to be used for food fortification purpose. In the first report, β-carotene-loaded NLCs were fabricated by the hot homogenization method and the physicochemical properties were evaluated [136]. Lacatusu et al. (2012) used a high-pressure homogenization method to encapsulate β-carotene in NLCs containing two natural oils (squalene and grape seed oil) [137]. The impact of the surfactant on physiochemical properties of NLCs encapsulating β-carotene was also studied [148]. Optimization of β-carotene encapsulation for NLCs using solvent diffusion methods was also carried out [337]. Similarly, β-carotene-loaded NLCs differing in the oil phase were also synthesized to evaluate the impact of the change in oil phase type on the physicochemical properties of the NLCs [297]. A high-pressure homogenization method was adopted to encapsulate 9Z-β-carotene and total β-carotene in NLCs for its physicochemical characterization and evaluated their stability during storage stability. It was observed that β-carotene-loaded NLCs stabilized both 9Z-β-carotene and total β-carotene not only from leakage but also from degradation against pH variations (pH 3.5, 4.5, 5.5, 6.5 and 7.5) and were found to be highly stable at 37°C over 21 days of storage [346]. Despite being the most advanced delivery method for processing of the sensitive bioactive compounds, applications of NLCs for β-carotene have been limited and their food applications are rather rare.

## 6. Safety Compliance and Risks of β-Carotene Nanoparticles

The customized properties of the discussed delivery systems, including the potential for bioavailability, better absorption and controlled release kinetics of the encapsulated bioactive compounds, may also impart unseen risks to biological systems [280,347]. It is assumed that utilization of biodegradable or natural materials may curtail the health hazards as compared to polymeric nanoparticles which are either derived from synthetic polymers or involve toxic organic solvents during their fabrication processes [347]. Due to the ambiguity on long- or short-term effects of direct or indirect employed nanoparticles in food systems, it is paramount to evaluate the impacts of nanoparticles on human health [348]. With regard to food safety, the FDA has listed certain strategies in conjunction with nanoparticle-based food and food components for mass production [349]. Regardless of the potential health concern, at present no standardized legislation for incorporation of nanoparticles in food systems, particularly for nanoparticles encapsulating β-carotene, are available. Nevertheless, several agencies and governmental bodies insist that we embrace the safety concerns of nanoparticle-based food products in legislative guidelines [350]. The European Food Safety Authority (EFSA) has published an excellent report on the topic (https://www.efsa.europa.eu/en/efsajournal/pub/5327, accessed on 20 December 2020). This guideline provides an overview on the required information about physico-chemical characterization and the other data requirements. It also states about the performance of risk assessment of nanomaterials in the food and feed area including novel food, FCMs, food/feed additives and pesticides. This lack of universal legislations compelled duty-bound policymakers to outline a guideline specifically dealing with the nanoscale materials in the food system [351].

The potentially tailored bioavailability of encapsulated bioactive compounds in delivery systems is a key safety concern, specifically for bioactive compounds, or the nanodelivery systems which may become toxic beyond a certain dose. To scrutinize the safety aspects, the bioavailability of bioactive compounds needs to be revaluated when it is encapsulated within nanodelivery vehicles, and reflections on alterations of the Recommended Daily Allowance (RDA) as well as the Tolerable Upper Intake Level (UL) of encapsulated bioactives are needed [352].

In addition, food scientists may also need to conduct studies addressing the safety concerns associated with nanoparticles, with special attention regarding: (i) the physiochemical characterization constraints of nanoparticles utilized in food items such as food additives, enzymes, flavorings, food contact materials (FCMs), novel foods, feed additives and pesticides [353]; (ii) development of the testing strategies to determine and characterize hazards transmitted via the engineered nanomaterials (ENMs)—i.e., assays for in vitro genotoxicity, absorption, distribution, metabolism and excretion and repeated-dose trials to study toxicity in test animals such as rodents [354].

In addition, the interactions between food items and nanodelivery systems should also be debated, which may result in producing radical oxygen species, photoreactions, etc. In December 2014, EU legislative bodies have insisted that food industries mention relevant information on the label if nano-food products are sold [351]. According to this guideline, particles have one or more dimensions of either 100 nm or less and agglomerates above 100 nm exhibiting ENM characteristics and should be considered as ENMs. In conjunction with this, the FDA has drafted guidelines which clearly define ENM-derived foods as (i) agents or products having particle sizes within the range of 1 to 100 nm with at least one dimension being within the nanoscale; (ii) agents or products exerting biological, chemical and physical characteristic associated with nanoscale materials and that are also on the nanoscale even though they are not nanosized.

In addition to legislative guidelines, there are several moral responsibilities of the food processing manufacturers, including: (i) evaluation of the changes imparted on the food materials—i.e., impurities and physiochemical properties; (ii) evaluation of the safety of food materials after modifications; (iii) submission of the regulatory assessment reporting to the legislative bodies such as FDA, FSSAI, EU, FASSAI, etc.; (iv) identification and a statement about the regulatory concern due to the ingestion of the nanoparticle-derived food items.

Apart from the US-FDA, several other regulatory authorities from various countries including Australia, New Zealand (FSANS) and Korea (MFDS) have issued their own guidelines [355]. These agencies counseled to conduct safety experiments (in vitro as well as in vivo) to evaluate the effect of nanoparticle-containing foods and publish the data, as well as to establish guidelines before releasing these nanoparticle containing foods to the food supply chain. Nevertheless, there is a lack of specific guidelines regarding nanoparticles containing foods, thus it is high time that the legislative bodies should come together to frame a more universal guideline for nanomaterial-derived food products which can then be applied or further tailored to different countries.

## 7. Fate of β-Carotene-Loaded Nanodelivery Systems

β-Carotene needs to be released in the GIT fluids to be taken up by the enterocyte for adsorption in GIT. The lipophilic nature of β-carotene limits its bioaccessibility to the cells due to the poor solubility. Lipid-based delivery systems, such as micelles, nano-/microemulsions, liposomes, niosomes, SLNs and NLCs, have recently been recognized as enhancing the bioaccessibility of many lipophilic vitamins and fat-soluble compounds including vitamins A, D and E [51,356,357,358,359]. The nature of the carrier oil utilized to fabricate LBDSs also affects their encapsulation efficiency and bioaccessibility [92,360].

The literature has also shown improved bioavailability of lipophilic bioactive compounds for various polymer-based delivery systems, such as micelles, nano-/microemulsions, hydrogel, nano-/microsphere, nano-/microcapsules and nanofibers [356,357]. However, a lack of dedicated comparative studies for various compounds on the use of such polymer-based delivery systems creates a research gap. Comprehensive, comparative and rigorous research is needed with the use of various delivery systems for each category of the compound to fill the research gap. Moreover, PBDS are well celebrated in pharmaceuticals to manipulate bioaccessibility by altering the solubility of β-carotene.

**Figure 4:**
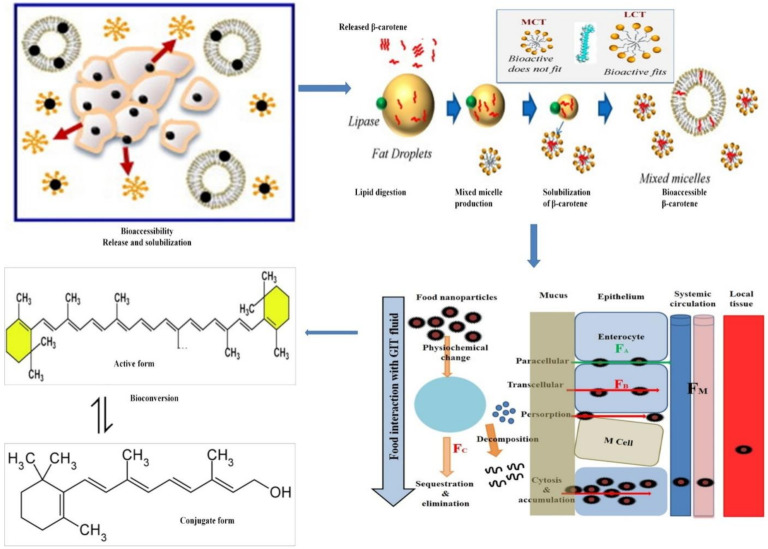
Factors influencing the bioavailability of β-carotene during absorption in the gastrointestinal tract (GIT). Paracelluar absorption, M-cell uptake via Peyer’s patches and Chylomicron-assisted enterocyte absorption.

Figure 4 illustrates the primary routes for β-carotene absorption in the small intestine. LBDSs have been primarily adopted to encapsulate lipophilic β-carotene and tend to enhance their absorption [87,181,195,228,229,242]. Mixed micelles produced as a result of digestion of LBDSs and penetrating through the aqueous mucous layer were created to make β-carotene bioaccessible to brush bordered enterocytes for uptake, while PBDSs undergo various digestive enzymatic alternations to release β-carotene from polymeric nanoparticles which are then absorbed by enterocytes. Further release of β-carotene from PBDSs and LBDSs and their packaging into chylomicrons inside the enterocytes is increased as a result of the high hydrophobicity [361,362]. These chylomicrons are lipid particles which are endogenously generated within the enterocytes using lipid components (free fatty acids, monoacyglycerols, and cholesterol) originating in part from mixed micelles produced as result of fat digestion [363]. Furthermore, these chylomicrons comprising β-carotene are then transported to the lymphatic circulation system to the liver for further processing.

Taken together, it is also believed that a fraction of encapsulated β-carotene from the delivery systems could be resistant to digestion and (i) may excrete from the GIT in undigested or semidigested condition or (ii) can penetrate the biological barriers of the intestine and enter the circulatory system [364]. The excretion of nanoparticles encapsulating β-carotene does not seem a viable approach; thus, such nanoparticles could not be a realistic commercial strategy for β-carotene encapsulation in the food sector as theycould poses unidentified health risks [365]. On the other hand, since nanoparticles could penetrate the biological barriers, the immunological and toxico-kinetic aspects of them need to be fully understood. Thus, it is advisable to carry out various investigations addressing the distribution of nanoparticles in cells and tissues, toxicological constraints and indecorous variation of nanoparticle properties. In summary, a suitable design for delivery system can overcome the safety hurdles to a great deal and the safety can be gauged in direct approaches. Furthermore, being cognizant about the full features of safety concerns is the key to a suitable design and to make nanodelivery systems commercially viable.

## 8. Conclusions

The selection of appropriated encapsulation techniques is the key for designing β-carotene delivery vehicles for food systems. Optimized doses of vital food components along with β-carotene, can be achieved using suitable delivery system and food items can be used as a platform for therapeutic as well as nutrient delivery.

Nanodelivery systems are the impending carriers for β-carotene. Moreover, solvent evaporation, solvent displacement, microfluidization, thin-film hydration and hot homogenization methods remain widely adopted encapsulation technologies for fabricating various β-carotene-nanodelivery systems. Each delivery system has its own technical complications that affect the final properties of the resulting nanodelivery system. However, the majority of ENMs have reported sustained release, high loading capacities, lower possibility of encapsulant expulsion, low toxicity and high encapsulant protection; however, these data are often extrapolated from pharmaceutical studies. Further studies in this domain are surely warranted for food enrichment purposes. More focused studies are required to obtain a better knowledge for the designing of delivery systems and to resolve the associated limitations such as the need for novel food grade polymers. Additionally, the safety of β-carotene delivery systems in food needs to be routinely investigated. This includes obtaining data from in vivo and in vitro studies involving all classes of available delivery systems. In summary, the stability of β-carotene delivery systems in food matrices as well as it’s delivery in the GIT need to be cautiously watched.

## 9. Future Prospects and Research Gaps

The great potential of delivery systems in food items is the new normal situation, which is becoming a routine. In light of the global health issues, these applications in food items seem imperious and indispensable to aid in combating diseases and promote healthy living. Several delivery systems have already been widely applied, including micro/nanoemulsions, NLCs, and PBDSs for food fortification of items such as ice creams and beverages. However, information regarding safety concerns associated with the incorporation of new ingredients and technologies must be generated by accelerated in vivo and clinical trials to support both policy makers and producers to provide the consumer with evidence-based information. Public acceptance of delivery system-based food is gradually improving, ensuring its huge potential in many ways, such as personalized nutrition with novel functionalities for evolving human physical and mental capabilities and improving mood and satisfaction from nano-based foods.

After a comprehensive review of the literature, the gaps in the existing literature were pointed and these research gaps should be addressed by future studies. The future research prospects recognized from existing literature on delivery system encapsulating β-carotene are as follows:

The field of designing nanodelivery systems for food applications is mainly trial and error-based. More interdisciplinary research needs be conducted to develop a set of universal methods for developing delivery systems that could display high compatibility towards β-carotene, target foods and their interaction with GIT fluids, cells, tissues and organs.

1. There is not a single report on the comparative assessment of the bioavailability of β-carotene EMS to the above mentioned nanodelivery systems. The data produced by devoted studies on bioavailability and health risks comparing various β-carotene-loaded delivery systems (LBDSs and PBDSs), particularly PBDSs, will aid in a better understanding and designing of suitable delivery systems for β-carotene.
2. The nature of the carrier oil (fatty acid chain length and degree of saturation) can also affect the biological fate of the lipid-derived delivery systems [366]. Nevertheless, data are too scarce with respect to LBDSs to draw a firm conclusion.
3. Although β-carotene-loaded delivery systems display a high bioavailability, other lipophilic compounds and related carotenoids may manipulate the bioavailability of β-carotene. More studies demonstrating the influences of lipophilic compounds present in the food matrix on the bioavailability of β-carotene-loaded delivery systems are needed. This will be aid in a better understanding in designing optimized delivery systems for β-carotene.
4. Many researchers have argued that nanoparticles may enhance the bioavailability of β-carotene due to the transfer of intact nanoparticles across enterocytes. Nevertheless, no single study witnessed the penetration of food grade nanoparticles containing β-carotene across intestinal walls in the available literature.
5. Most of the delivery systems are fabricated based on extrapolated in vitro and in vivo pharmaceutical data. This cannot be applied to food grade nanoparticles—in particular, polymer-based delivery systems.
6. Certain ingredients (EDTA, chitosan, fatty acid, etc.) can manipulate the structure and integrity of the cell membrane. This is perhaps the least explored field and data generated on the effect of these ingredients on the cell membrane are necessary for better understanding and designing efficient β-carotene delivery systems.
7. Various research studies displayed the improved permeability of cell membranes for certain kind of nanoparticles. Most of these are coupled with pharmaceutical formulations, containing to some extent certain nonfood grade materials. The same conclusion cannot be drawn for food grade nanoparticles. Thus, more devoted and rigorous investigations are needed to evaluate the impact of food grade nanoparticles on the penetration of cell membranes.
8. There is ambiguity on interactions between GIT fluids and nanoparticles encapsulating β-carotene. It is sensible to debate how the bioavailability of β-carotene is influenced when it is encapsulated in available delivery systems.
9. The perceived risks endorsed within the transfer of intact particles across the intestinal walls into the systemic circulation and buildup of particles or β-carotene in organs and the incidence of very high peak concentrations of β-carotene in the blood is poorly understood. Since reliable data signifying toxicity or risks are not present in the current literature, this should spark a debate on the various unknown factors.
10. The role of digestive enzymes in the release of β-carotene from delivery system as well as on its bioavailability is not fully known. The assessment addressing the effects of enzymes individually or in arrays and their concentration on the bioavailability of β-carotene from delivery systems will aid in better knowledge for designing suitable delivery system.
11. There is ambiguity regarding the kinetics of nanoparticle transfer from food matrix GIT fluid as well as from GIT fluid to enterocytes. More data need to be generated to understand the transfer kinetic of nanoparticles, which will result in a better understanding toward the realization of better delivery systems of β-carotene for food applications.

## Figures and Tables

**Table 1:** Engineered nanoparticle-based delivery systems for enhancing the bioavailability of β-carotene.

Class of Delivery Systems	Subclass of Delivery System	Delivery System	Ingredients	Technique/Preparation Method	Physiochemical Studies	Encapsulation Efficiency	Release Studies	Particle Size	Cellular/Animal Studies	Applications	References
Lipid-derived delivery systems	Self-assembled delivery system	Liposome	Hydrogenated soybean PC Lipoid	Ethanol injection method	FTIR, SEM, Raman microspectroscopy, UV-vis irradiation	NA	NA	NA	NA	NA	[117]
			PC	Dehydration/rehydration method	NA	NA	NA	NA	Microsomes/Rat	pharmaceutical	[118]
			Hydrogenated soy PC Lipoid GmbH Xanthan gum	Spray-drying	DSC, small-angle X-ray scattering (SAXS), TEM, DLS, ELS	NA	NA	700–3000 nm	NA	NA	[119]
			Gama-oryzanol	Modified thermal method	FTIR	NA	NA	64–500 nm	NA	NA	[120]
			PC PS PEA	Dehydration/rehydration method	NA	NA	NA	NA	Hamster	pharmaceutical	[121]
			Phospholipids (Lipoid S-100-H and Lipoid S-40, Lipoid GmbH) Sucrose	Spray-drying	DLS, ELS, XRD, SEM	NA	NA	285–1695 nm	NA	NA	[122]
			Egg yolk phospholipid Tween 80	Thin-film evaporation method	DLS, AFM	NA	SGF, SIF	600 nm	NA	NA	[123]
		Niosome	Spans 40, 60, 80 Tween 20, 40, 60 Cholesterol	Dehydration/rehydration method	DLS, EE, TEM	16.0–51%	NA	273.2–367.9 nm	RAT-1 immortalized fibroblasts	pharmaceutical	[124]
	Particulate delivery systems	Solid lipid nanoparticles	Hydrogenated canola stearin Polyoxyethylene Sorbitan monolaurate	Hot homogenization	DLS, DSC, ELS, Cryo TEM, NMR, XRD	NA	NA	111.7–170.8 nm	NA	NA	[125]
			SC WPI SPI	Microfluidization	DLS, TEM	99.1%,98.8%,	NA	77.8–190.9 nm	Caco-2 cells	NA	[126]
			Tripalmitin Phospholipid Polyethylene glycol sorbitan monooleate	Hot high-pressure homogenization	DLS, ELS, DSC	NA	NA	0.16–0.27µm	NA	NA	[127]
			WPI Corn oil	Homogenization	DLS, ELS, TEM, SEM	NA	NA	<200 µm	NA	NA	[128]
			SPI Xanthan gum Palm stearin	Hot homogenization	DLS	NA	NA	1.20–1.70 µm	NA	Food application (ice creams)	[129]
			Brij 30 Octadecane	Phase-inversion temperature	DLS, DSC	NA	NA	109 and 128 nm	NA	NA	[130]
			Tristearin Sunflower oil Hydrogenated soy lecithin Tween 80	Hot pressure homogenization	DLS, ELS, DSC	NA	NA	NA	NA	NA	[131]
			Hydrogenated palm oil Cocoa butter Tween 20	Hot high-pressure homogenization method	DLS, DCS, NMR	NA	NA	168–227 nm	NA	NA	[132]
			Polyoxyethylene Tween 80	Phase-inversion temperature	AFM, DLS, DSC, XRD	NA	NA	<400 nm	NA	NA	[133]
			Stearic acid Sunflower oil Tween 80	Hot agitation	DLS, DSC, XRD	NA	NA	<5 µm	NA	NA	[134]
		Nanostructured lipid carriers	Glyceryl tristearate High oleicsunflower oil Tween 80	Solvent displacement technique	DLS, DSC	NA	NA	500 nm	NA	NA	[135]
			Propylene glycol monostearate Propylene glycol mono- and distearates Propylene glycol mono- and dipalmitates Sunflower oil	Hot homogenization	DLS, DSC	NA	NA	82–217 nm.	NA	NA	[136]
			Tween 80 Tween 60 Tween 80 Phosphatidylcholine Grape seed oil	Hot homogenization	DLS, ELS, DSC, TEM	65.26–74.35%	NA	85.2–129.2 nm	NA	NA	[137]
			Cremophor RH40 Span 80 Cupuacu butter	Phase-inversion temperature	DLS, DSC, TEM	NA	Gastric fluid, Duodenal fluid, Jejunal fluid, Ileal fluid	31.6–34.08 nm	NA	NA	[138]
		Microemulsion	Span 80 Span 40 Tween 80 virgin coconut oil Palm oil	Spontaneous emulsification method	DLS, ELS	NA	NA	20–22.60 nm	NA	NA	[139]
			Lactoferrin Β-Lactoglobulin	Microfluidization	DLS, ELS	NA	NA	<250 nm	NA	NA	[140]
			Sucrose monolaurate Lactoglobulin Whey proteins	Microchannel Device	DLS	NA	na	27.9 µm	NA	NA	[141]
			Hydrogenated canola stearin Tween 20	Hot homogenization	DLS, DSC, ELS, Cryo TEM, NMR, XRD	NA	na	115 nm	NA	NA	[125]
			Tween 20 Corn oil	Microfluidization	DLS, CFFM	NA	SSF, SGF	0.21–23 µm	NA	NA	[142]
		Nano emulsion	Corn oil Lemon oil Sucrose Ponopalmitate Lysolecithin	Microfluidization	DLS	NA	SSF, SGF, SIF	<150 nm	NA	NA	[91]
			Long-chain triglyceride Medium-chain triglyceride Tween 20	Microfluidization	DLS	NA	SSF, SGF, SIF	140–170 nm	NA	NA	[92]
			Corn oil	Hot homogenization	DLS	NA	SSF, SGF, SIF	<200 nm	NA	NA	[143]
			MCT oil	Microfluidization	DLS, ELS	NA	NA	97.2–416.0 nm	NA	NA	[144]
			Tween 80	Supercritical fluid	DLS	NA	NA	50–150 nm	NA	NA	[145]
			Corn oil Tributyrin	Homogenization	DLS	NA	SSF, SGF, SIF	1.25–1.34 µm	NA	NA	[146]
			Tween 80 Stearic acid	High-speed homogenization	DLS	NA	NA	418.8–1689.0 nm	NA	NA	[147]
			Tween 20 Corn oil	Microfluidization	DLS, DSC	NA	SSF, SGF, SIF	0.2–23 µm	NA	NA	[142]
			Compritol Poloxamer 407	Hot-high shear homogenization	DLS, ELS	NA	NA	79–115 nm	NA	NA	[148]
			Miglyol 812 (MCT) Corn oil (LCT)	Microfluidization	DLS, ELS, DSC	NA	SSF, SGF, SIF	146 to 415 nm,	NA	NA	[149]
			Sunflower lecithin Tween 20 Peppermint oil	Heating and stirring	DLS	NA	NA	<10 nm	NA	NA	[150]
			Orange oil Β-lactoglobulin Tween 20	Microfluidization	DLS	NA	NA	<100 nm	NA	NA	[151]
			Miglyol-812 (caprylic/capric triglycerides	Spontaneous emulsionfication method	DLS, SEM	NA	NA	100–300 nm	NA	NA	[152]
Polymer-derived delivery systems	Self-assembled polymer-derived delivery systems	Starch-based emulsion	NaCMC Kappa-carrageenan	Cross-linking	SEM	NA	NA	700 nm	NA	NA	[153]
			Medium-chain triacylglycerol MCT oil OSA-modified starches	Spray-drying	DLS, ELS, SEM	NA	NA	114–118 nm,	NA	NA	[154]
			Lactoferrin Β-Lactoglobulin	Microfluidization	DLS, ELS	NA	NA	208–385 nm	NA	NA	[140]
			Modified starches	High-pressure homogenization	DLS	NA	SGF, SIF	17 nm	NA	NA	[155]
			OSA-starch	Ultrasound emulsification	SEM	NA	NA	300–600 nm	NA	NA	[156]
			SSPS Beetpectin	Layer-by-layer electrostatic deposition method	DLS, ELS	NA	NA	250.0–306.3 nm 304.5–466.6 nm	NA	NA	[157]
			OSA-modified starch MCT	Microfluidization	DLS	NA	SGF, SIF	80.0 ± 1.3 nm	NA	NA	[158]
		Protein-based emulsion	SC WPC	Solvent-displacement method	DLS, ELS	NA	NA	45–127 nm	NA	NA	[159]
			SC	Spontaneous emulsification	DLS, SEM	100 ± 1%	NA	50–500 nm	NA	NA	[160]
			A-lactalbumin Catechin	Microfluidization	CD, DLS, ELS	NA	NA	158.8 and 162.7 nm	NA	NA	[161]
			Protein powders Sucrose syrup	Homogenization	DLS, DSC	NA	NA	0.48–0.66 µm	NA	NA	[162]
			Sunflower oil Hydrogenated palm kernel oil WPI SC	High speed homogenization	DLS, XRD	NA	NA	0.46–0.50 µm	NA	NA	[163]
			WPI	pH-cycling method	DLS, ELS, FTIR, SEM	NA	SGF, SIF	409.7 nm	NA	NA	[164]
			Beta-lactoglobulin Catechin	Microfluidization	DLS, ELS	NA	NA	160–170 nm	NA	NA	[165]
			WPI sunflower oil Gum arabic	Layer-by-layer electrodeposition technique	DSC, Dynamic Mechanical Analyses (DMA)	NA	NA	NA	NA	NA	[166]
			SC Corn oil	Microfluidization	DLS	NA	NA	124–368 nm	NA	NA	[167]
			SC Tween 20	Solvent displacement technique	DLS, ELS	NA	NA	30–206 nm	NA	NA	[168]
			Lactoferrin MCT	Homogenization	CD, DLS, ELS, FSS (Fluorescence spectroscopy)	NA	NA	302–583 nm	NA	NA	[169]
			SC Alginic acid	Microfluidization	DLS, ELS, FSS	NA	SGF, SIF	0.48–1.87 µm	NA	NA	[170]
			Corn oil Canola oil Olive oil SC	Microfluidization	DLS	70.9%	SGF, SIF	167.4–178.8 nm	Caco-2,Cell toxicity	Pharmaceutical	[171]
		Carbohydrate-based emulsion	SA Tween 80	Sonication and hot homogenization	DLS, ELS, CFSM	NA	SSF, SGF, SIF	0.2–23 µm	NA	NA	[172]
			Mannitol Gelatin	Freeze-dryer	DSC	NA	NA	NA	NA	NA	[173]
		Micelle	SC Whey protein hydrolysate	Solvent displacement	DLS, ELS, FSEM	NA	NA	13–171 nm	NA	NA	[174]
			SC	Spontaneous emulsification	DLS, SEM	100 ± 1%	NA	50–500 nm	NA	NA	[160]
			Hydroxyethyl cellulose Lionic acid	Sonication	DLS, FTIR, NMR, SEM, TEM	84.67%	SSF, SGF, SIF	20–50 nm	NA	NA	[175]
			Casein	Microfiltration	DLS, FTIR, TEM	NA	NA	0.04–0.4 µm	NA	NA	[176]
			Chitosan PLA	Polymerization	DLS, FTIR, NMR, XRD, TEM	NA	NA	14 nm	NA	NA	[177]
			Soybean oil Tween 20 Tween 40 Tween 80 Glycerol monocaprylocaprate Propylene glycol dicaprylate/dicaprate Caprylic/capric triglyceride	Homogenization	DLS, ELS, TEM	NA	NA	12–100 nm.	Caco-2, Cell toxicity study	Food application	[178]
			PLA Tween 80	Solvent displacement method	DLS, ELS	NA	NA	0.087–1.158 µm	NA	NA	[179]
	Particulate nanoparticles	Molecular complex	Γ-cyclodextrin	Co-precipitation and physical mixture techniques	FTIR, FESEM	NA	NA	NA	NA	NA	[113]
			Sunflower seed oil acacia gum Maltodextrin	Spray-drying	SEM	NA	PBS	NA	NA	NA	[115]
			Amylose	Sonication	DLS, TEM, SEM, XRD	65%	NA	12 ± 3 nm	NA	NA	[116]
		Nanosphere	PLA Stearyl amine Stearoyl polyoxyl-32 glycerides	Nanoprecipitation method	DLS, ELS	NA	PBS	117.1 ± 4.6 nm	MCF-7 breast cancer cells, Cell toxicity studies	Pharmaceutical	[180]
			Rice protein isolate	Homogenization	CD, DLS, FTIR, CLSM	NA	SGF, SIF	300−400 nm	NA	NA	[181]
			Zein	Microfluidization	DLS, ELS, TEM	NA	SGF, SIF	32.44 ± 0.87–168.17 ± 22.36 nm	NA	Food application (milk)	[182]
			Sunflower oil WPI Trehalose Gum Arabic	Microfluidization	DLS, ELS, Raman-FIB-SEM	NA	NA	46.77 ± 0.17–48.23 ± 0.13 µm	NA	NA	[183]
			Corn starch	Nanoprecipitation method	DLS, DSC, XRD	NA	SIF	0.77–0.89 µm	NA	NA	[184]
			Poly[poly(oxyethylene-1500)-Oxy-5- dodecanyloxyisophthaloyl Poly [poly-(oxyethylene-1500)-oxy-5- hydroxyisophthaloyl]	Homogenization	DLS, SEM, TEM, NMR	22.60–28.08%	Water, Buffer	<100 nm	NA	NA	[185]
			OSA -modified starches OSA-dextrin	High-temperature, high-pressure emulsification and antisolvent precipitation	DLS	70–80%	NA	137–135,900 nm	NA	NA	[186]
			SC WPI SPI	Homogenization-evaporation method	DLS, DSC, ELS, FTIR, XRD	NA	SGF, SIF	NA	Caco-2 cells	NA	[187]
		Microsphere	OSA-modified starches OSA-dextrin	Precipitation	DLS, SEM	65–90%	NA	300–600 nm	NA	NA	[188]
			Κ-carrageenan Oil	Ionic gelation	DLS	NA	SGF, SIF	80–94 nm, 91–106 nm, 128–134 nm	NA	NA	[189]
			WPI Dextran	Glycosylation conjugation	CD, DLS, ELS	NA	SGF, SIF	165.6–176.0 nm	NA	NA	[190]
			Hydroxypropyl methylcelluloses Kosher gum acacia	High pressure homogenization	DLS	NA	NA	1.38–1.96 mm.	NA	NA	[191]
			SC Arabic gum	Electrostatic complexation	DSC, FTIR	NA	NA	NA	NA	NA	[192]
			Shellac	Syringe microfluidization	SEM	NA	NA	19–84 µm	NA	NA	[193]
			Casein Maltodextrin	Microfluidization and Spray-drying	DLS, ELS	NA	NA	230–277 nm	NA	NA	[194]
			Canola oil Ethylcellulose	Ionic gelation	Lipid lipolysis	NA	NA	NA	NA	NA	[195]
			SPI	Freeze-drying	AFM DLS, ELS	NA	SGF, SIF	55 nm	NA	NA	[196]
			Poly (methyl methacrylate)	Spontaneous emulsification	DLS	14.18–64.39%	NA	655–3418 nm	NA	NA	[197]
			PLA	Electrospinning	SEM	NA	NA	NA	NA	NA	[198]
			Casein	Microfiltration	DLS, FTIR, TEM	NA	NA	0.04–0.4 µm	NA	NA	[176]
			Almond gum Gum Arabic	Spray-drying and freeze-drying	DLS	66–70%	Sunflower oil	1.20–2.30 µm	NA	NA	[199,200]
			Almond gum Gum Arabic	Freeze-drying	DLS	66–70%	Sunflower oil	2.10–3.2 µm	NA	NA	[200]
			Caseins	Spray-drying	Photodegradation study	NA	NA	NA	NA	NA	[201]
			OSA-modified starches Flax seedoil	Microfluidization	DLS, ELS, FESEM	90%	NA	165.0–129.1 nm	NA	NA	[202]
			Casein Dextran	Dry heating method	DLS, DSC, FSM	73.64–74.53	SGF, SIF	111.1–127.3 nm	NA	NA	[203]
			WPI Corn oil	Microfluidization	DLS, ELS	NA	NA	0.14–0.16 μm	NA	NA	[204]
			MCT coconut oil Corn oil span 20 Monostearin	High pressure homogenization	DLS	NA	NA	176.3–228 nm	CACO-2 CELLS, RATS	PHARMACEUTICAL AND FOOD	[205]
			WPI SC	High-pressure homogenization	DLS, ELS	NA	SGF, SIF	142 ± 6–160 ± 10 nm	CACO-2 CELLS	NA	[206]
			SC Maltodextrin	High-pressure homogenization	DLS, LD, TEM	NA	NA	262.8 ± 4.10–307.1 ± 5.40 nm	NA	NA	[207]
			Xanthan Gum Palm stearin Hydrolyzed SPI	Homogenization	DLS, DSC, ELS, FFS	NA	NA	1–1.5 µm	NA	NA	[208]
			Chitosan	Cross-linking and sonication	DLS, SEM	NA	NA	1570.0 nm.	NA	Food application (hamburger patties)	[209]
			Soybean oil Ulva fasciata polysaccharide	Microfluidization	DLS	NA	SSF, SGF, SIF	0.82 µm	NA	NA	[210]
			Zein Carboxymethylchitosan	Rotating evaporation	DLS, DSC, ELS, FTIR, SEM	56.5–92.7%	SGF, SIF	70.41 ± 0.67–420.9 ± 2.34 nm	NA	NA	[211]
			OSA-modified starch Tween-80 Flax seed oil MCT	Microfluidization	DLS, ELS	NA	NA	123.9–207.2 nm	NA	NA	[212]
			Flax seed oil MCT OSA modified starch Tween-80	Microfluidization	DLS, ELS	NA	NA	123.9–207.2 nm	NA	NA	[212]
			Carrageenan Tween 20 and 80	Polymerization	DLS	NA	SGF, SIF	127–149 nm	NA	NA	[213]
			Casein Guar gum	Homogenization and coacervation pr	DLS, ELS, FTIR, SEM	65.95 ± 5.33%	SGF, SIF	176.47± 4.65 µm	NA	NA	[214]
			Egg protein	High-pressure homogenization	DLS	NA	NA	10.1 ± 0.7–14.5 ± 0.6 nm	NA	NA	[215]
			Tween 20 Corn oil Sucrose	High-pressure homogenization	DLS	NA	SGF, SIF	170 nm	NA	NA	[216]
			Soybean oil WPI	High pressure homogenization	Effect of digestion on particle size	NA	SSF, SGF, SIF	NA	NA	NA	[217]
			Maltodextrin Gum arabic Gelatin	Spray-drying	Stability of carotene in powder	NA	NA	NA	NA	Food application	[218]
			Poly(D, L-lactide-co-glycolide)	Solvent evaporation	DLS	14%	NA	260 nm	NA	Pharmaceutical	[219]
			Calcium caseinate SA	Homogenization and sonication	DLS, SEM	79.63 ±1.41–84.32 ± 1.08%	SGF,	210.5 1.23 nm	NA	NA	[220]
			Soybean-soluble polysaccharides Chitosan	Homogenization	DLS, ELS	NA	NA	0.52 µm.	NA	NA	[221]
			Poly-(3-hydroxybutyrate-co-3-hydroxyvalerate)	Supercritical carbon dioxide micronization technique	NA	NA	organic solvent	NA	NA	NA	[222]
	Capsular nanoparticles	Microcapsule	Maltodextrin Tween 80	Freeze-drying	CFLM, DLS, ELS	NA	SGF, SIF	0.23 ± 0.02–0.24 ± 0.01 µm	NA	NA	[115]
			Hydrolyzed starch	Homogenization	Stabilitystudy	NA	NA	NA	NA	NA	[223]
			Arabic gum	Spray-drying	NA	NA	NA	NA	NA	NA	[224]
			Medium-chain triacylglycerol MCT	High speed homogenization, spray-drying	DLS, SEM	NA	NA	114–159 nm	NA	NA	[154]
			Gum arabic	Spray-drying	DLS, SEM	NA	NA	19.69–20.98 µm	mouse bone marrow and peripheral blood cells/Wistar albino rats,	Pharmaceuticals	[225]
			Poly-ɛ-caprolactone	Emulsification–diffusion method	DLS, ELS, SEM	NA	NA	250–650 nm.	NA	NA	[226]
			Casein Gum Tragacanth	Complex coacervation	CLSM, DLS, FTIR, SEM, TGA, XRD	79.36±0.541%	SGF	159.71±2.16 µm	NA	NA	[227]
			Chitosan SA	Spray-drying	DLS	34–55%	SIF	852–958 μm	NA	NA	[228]
			Soybean oil SPI	Homogenization	CLSM	NA	NA	0.23 ± 0.02–6.68 ± 0.65 lm	NA	NA	[229]
			Poly(hydroxybutirate-co-hydroxyvalerate)	Supercritical fluid	SEM	NA	NA	NA	NA	NA	[230]
			Poly(hydroxybutirate-co-hydroxyvalerate)	Supercritical fluid	SEM	0.95–55.54%	NA	1.3–51.9 µm	NA	NA	[231]
			Chitosan Oleic acid Fe3O4	Solvent displacement technique	SEM, XRD	78.74–81.2%	PBS	NA	NA	NA	[232]
			Dextrin	Precipitation	DLS, DSC, TEM, XRD	NA	SGF	16–30 nm	NA	NA	[233]
			Gum arabic Gelatin Maltodextrin	Freeze-dryer	DSC	NA	NA	NA	NA	NA	[234]
			Oil Tween 20	Homogenization and evaporation	CFLS, DLS	NA	NA	161.98 ± 17.19–189.45 ± 22.69 nm	NA	NA	[235]
			WPC Tween 20	Membrane emulsification	DLS	NA	NA	1.28 ± 0.02–1.69 ± 0.49 µm	NA	NA	[236]
			Pea protein concentrate Maltodextrin	Spray-drying	DLS, SEM	NA	Water	4.9 + 2.4–6.0 + 3.0 µm	NA	NA	[237]
			Lactose Trehalose	Spray-drying	DLS, DSC	NA	NA	0.2–0.8 µm	NA	NA	[238]
		Nanocapsule	Poly-ɛ-caprolactone	Emulsification–diffusion method	DLS, ELS, SEM	NA	NA	250–650 nm	NA	NA	[226]
			Poly-ε-caprolactone polymer Tween 80 Triglycerides of the capric and caprylic acids	polymer method (Nanoinjection and stirring)	DSC, ELS, TEM	99.65–99.75%	NA	142.33–190.33 nm	NA	NA	[239]
			Lecithin Tween20	Homogenization and ultrasonication	DLS, DSC, SEM, XRD	2.23±1.42%	PBS	255.9±1.63 nm	NA	NA	[240]
	Fibrous nanoparticles	Nanofiber	Polyethylene	Electrospinning	DSC, FTIR, SEM	NA	NA	NA	NA	MA	[241]
			Maltodextrin Alginate Chitosan	Spray-drying	DLS, SEM	NA	SSF, SGF, SIF	10.5–942.8 µm	NA	Food application	[242]
			PLA	Electrospinning	SEM	NA	NA	NA	NA	MA	[198]
		Nanotube	PVA Polyethylene oxide	Electrospinning	FTIR, SEM, RSM	NA	NA	250 nm	NA	NA	[117]
	Gelatinous nanoparticles	Hydrogel	SA Calcium alginate	Freeze-drying	SEM	NA	PBS	NA	NA	NA	[115]
			Sodium carboxymethyl cellulose Kappa-carrageenan	Cross-linking	SEM	NA	SGF	NA	NA	NA	[153]
			SC	Solvent-displacement method	DLS, ELS	NA	NA	45–127 nm	NA	NA	[159]
			WPI Alginic acid	Microfluidization	CFLS, DLS, ELS	NA	SSF, SGF, SIF	285–660 mm	NA	NA	[243]
			Rice starch Xanthan gum WPI	Microfluidization	CFSL	NA	SSF, SGF, SIF	450 nm	NA	NA	[244]
			Ethylcellulose Canola oil	Heating and stirring	Bioaccessibility	NA	SSF, SGF, SIF	NA	NA	NA	[245]
			Pea protein isolate Sunflower oil	Microfluidization	DLS	NA	SSF, SGF, SIF	3.16–22.1 µm	NA	NA	[246]
			Codium alginate Δ-glucono-lactone Tween 80	Spontaneous emulsification	Bioaccessibility, DLS	NA	SSF, SGF, SIF	79–138 nm	NA	NA	[247]
			WPI	Ultrasonic emulsification	CFSL, DLS, ELS	NA	SGF	78–252 nm	NA	NA	[248]
			Soy glycinin	Microfluidization	CFLS, DLS	NA	NA	1.5–9.7 µm	NA	NA	[249]
			Corn oil, WPI Rice starch	Hot homogenization	CFSL, Bioaccessibility	NA	SSF, SGF, SIF	NA	NA	NA	[250]

NA: not applicable, AFM: atomic force microscopy, CFM: confocal fluorescent microscope, CLSM: confocal laser scanning microscopy, DLS: dynamic light scattering (used for size determination), DSC: differential scanning calorimetry, EE: encapsulation efficiency, ELS: electrophoretic light scattering (used for zeta potential determination), FRF: fractional residual fluorescence, FSM: fluorescence spectrophotometer, FTIR: Fourier transform infrared spectroscopy, NMR: nuclear magnetic resonance, PBS: phosphate buffered saline, SEM: scanning electron microscope, SGF: simulated gastrointestinal fluid, TEM: transmission electron microscope, XRD: X-ray diffraction, FSP: Florescence spectrophotometry, CM: confocal microscopy, FRF: fractional residual fluorescence, SRB: cellular proliferation assay (colorimetric) and MTT: cellular viability assay (colorimetric).

**Table 2:** Various factors that need to be considered prior to selecting a delivery system for encapsulating any bioactive agent.

ENMS	Class of DeliverySystem	Subclass of Delivery System	Ability to Deliver Lipophilic and Lipophobic BA	Physical Stability	Biological Stability	Biocompatibility	Drug Targeting	Drug Loading	Feasibility to be Delivery System for β-Carotene
Lipid-derived delivery system	Self-assembled delivery system	Liposome	Yes	poor	Poor	Good	Moderate	Low to moderate	Poor
		Niosome	Yes	moderate	Poor	Moderate	Moderate	Moderate	Poor
	Particulate	Solid lipid nanoparticles	Only lipophilic	Good	Moderate	Good	Moderate	Moderate	Moderate
		Nanostructured lipid carriers	Only lipophilic	Good	High	Good	Moderate	High	Good
	Emulsion	Microemulsion	Yes	Moderate	Moderate	Good	Poor	High	Good
		Nanoemulsion	Yes	poor	Moderate	Good	Poor	High	Poor
Polymer-derived delivery system	Self-assembled delivery system	Starch-based Micelle	Yes	Good	Good	Moderate	Poor	Poor	Good
		Protein-based micelles	Yes	Poor	Good	Moderate	Moderate	Poor	Good
		Carbohydrate							Poor
		Hydrogel	Yes	Good	Good	Poor	Poor	Poor	Good
		Colloidal nanoemulsion	Yes	Moderate	Moderate	Good	Poor	High	moderate
		Nanoemulsion	Yes	poor	Moderate	Good	Poor	High	Poor
		Molecular complexes	Only lipophilic	Good	Moderate	Poor	Poor	Low	Poor
	Particulate	Protein inclusion complexes	Yes	Good	Moderate	Moderate	Moderate	Low	Poor
		Nanosphere	Yes	Good	Moderate	Moderate	Moderate	Moderate	Poor
		Microsphere	Yes	Good	Moderate	Moderate	Moderate	Low	Moderate
	Fibrous	Nanofiber	Yes	Good	Moderate	Moderate	Moderate	Low	Poor
	Capsular	Microcapsule	Yes	Good	Moderate	Moderate	Moderate	Low	Poor
		Nanosphere	Yes	Good	Moderate	Moderate	Moderate	Moderate	Poor

## Abbreviations

Cremophor	40 PEG hydroxylated castor oil
EC	Ethylcellulose
GA	Gum arabic
LCT	Long-chain triglyceride
MCT	Medium-chain triacylglyceride
NaCMC	Sodium carboxymethyl cellulose
OSA	N-octenyl succinic anhydride
PC	Phosphatidylcholine
PEA	Phosphatidylethanolamine
PHBV	Poly(hydroxybutirate-co-hydroxyvalerate)
PLA	Poly(lactic) acid
PS	Phosphatidylserine
PVA	Polyvinyl alcohol
SA	Sodium alginate
SC	Sodium caseinate
SGF	Simulated gastric fluid
SIF	Simulated intestinal fluid
SPI	Soy protein isolate
SSF	Simulated saliva fluid
SSPS	Soybean-soluble polysaccharides
Tween	Polyoxyethylenesorbitan monolaurate
WPC	Whey protein concentrate
WPI	Whey protein isolate

## References

[1] McLaren D.S., Kraemer K. (2012). Manual on Vitamin A Deficiency Disorders (VADD).

[2] Unit N., World Health Organization (1995). Global Prevalence of Vitamin A Deficiency.

[3] Rodriguez-Concepcion M., Avalos J., Bonet M.L., Boronat A., Gomez-Gomez L., Hornero-Mendez D., Limon M.C., Meléndez-Martínez A.J., Olmedilla-Alonso B., Palou A. (2018). A global perspective on carotenoids: Metabolism, biotechnology, and benefits for nutrition and health. Prog. Lipid Res..

[4] Shibata M., Sato H., Shimizu T., Shibata S., Toriumi H., Kuroi T., Ebine T., Iwashita T., Funakubo M., Akazawa C. (2013). Differential cellular localization of antioxidant enzymes in the trigeminal ganglion. J. Headache Pain.

[5] Adwas A.A., Elsayed A., Azab A.E., Quwaydir F.A. (2019). Oxidative stress and antioxidant mechanisms in human body. J. Appl. Biotechnol. Bioeng..

[6] Giustarini D., Dalle-Donne I., Tsikas D., Rossi R. (2009). Oxidative stress and human diseases: Origin, link, measurement, mechanisms, and biomarkers. Crit. Rev. Clin..

[7] Adam-Vizi V., Chinopoulos C. (2006). Bioenergetics and the formation of mitochondrial reactive oxygen species. Trends Pharmacol. Sci..

[8] Lenaz G. (2012). Mitochondria and reactive oxygen species. Which role in physiology and pathology?. Adv. Mitochondrial. Med..

[9] Auten R.L., Davis J.M. (2009). Oxygen toxicity and reactive oxygen species: The devil is in the details. Pediatr. Res..

[10] Sharifi-Rad M., Anil Kumar N.V., Zucca P., Varoni E.M., Dini L., Panzarini E., Rajkovic J., Tsouh Fokou P.V., Azzini E., Peluso I. (2020). Lifestyle, oxidative stress, and antioxidants: Back and forth in the pathophysiology of chronic diseases. Front. Physiol..

[11] Engwa G.A., Ferdinand P.U., Nwalo F.N., Unachukwu M.N. (2019). Mechanism and health effects of heavy metal toxicity in humans. Poisoning in the Modern World-New Tricks for an Old Dog?.

[12] Leni Z., Künzi L., Geiser M. (2020). Air pollution causing oxidative stress. Curr. Opin. Toxicol..

[13] Klawitter J., Klawitter J., Pennington A., Kirkpatrick B., Roda G., Kotecha N.C., Thurman J.M., Christians U. (2019). Cyclophilin D knockout protects the mouse kidney against cyclosporin A-induced oxidative stress. Am. J. Physiol. Ren. Physiol..

[14] Wu S.-C., Yen G.-C. (2004). Effects of cooking oil fumes on the genotoxicity and oxidative stress in human lung carcinoma (A-549) cells. Toxicol. In Vitro.

[15] Bardaweel S.K., Gul M., Alzweiri M., Ishaqat A., Alsalamat H.A., Bashatwah R.M. (2018). Reactive oxygen species: The dual role in physiological and pathological conditions of the human body. Eurasian J. Med..

[16] Chen Q., Wang Q., Zhu J., Xiao Q., Zhang L. (2018). Reactive oxygen species: Key regulators in vascular health and diseases. Br. J. Pharmacol..

[17] Franchina D.G., Dostert C., Brenner D. (2018). Reactive oxygen species: Involvement in T cell signaling and metabolism. Trends Immunol..

[18] Martina A., Jana P., Anna S., Tomas B. (2012). Nitric oxide—Important messenger in human body. J. Mol. Integr. Physiol..

[19] Hezel M.P., Weitzberg E. (2015). The oral microbiome and nitric oxide homoeostasis. Oral Dis..

[20] Belhadj Slimen I., Najar T., Ghram A., Dabbebi H., Ben Mrad M., Abdrabbah M. (2014). Reactive oxygen species, heat stress and oxidative-induced mitochondrial damage. A review. Int. J. Hyperth..

[21] Kaulmann A., Bohn T. (2014). Carotenoids, inflammation, and oxidative stress—implications of cellular signaling pathways and relation to chronic disease prevention. Nutr. Res..

[22] Kawata A., Murakami Y., Suzuki S., Fujisawa S. (2018). Anti-inflammatory activity of β-carotene, lycopene and tri-n-butylborane, a scavenger of reactive oxygen species. In Vivo.

[23] Evans R.M., Mangelsdorf D.J. (2014). Nuclear receptors, RXR, and the big bang. Cell.

[24] Palczewski G., Widjaja-Adhi M.A., Amengual J., Golczak M., Von Lintig J. (2016). Genetic dissection in a mouse model reveals interactions between carotenoids and lipid metabolism. J. Lipid Res..

[25] Krinsky N.I., Johnson E.J. (2005). Carotenoid actions and their relation to health and disease. Mol. Aspects Med..

[26] Czarnewski P., Das S., Parigi S.M., Villablanca E.J. (2017). Retinoic acid and its role in modulating intestinal innate immunity. Nutrients.

[27] Al Binali H.A.H. (2014). Night blindness and ancient remedy. Heart Views.

[28] Biradar S.T., Ankitha C.S., Pasha S.M. (2020). An Ayurvedic insight to keratoconus-A case report. J. Ayurveda Integr. Med..

[29] Balaji S., Roy A. (2020). Beta-carotene--A versatile antioxidant in oral cancer: A review. Drug Invent. Today.

[30] Miller A.P., Coronel J., Amengual J. (2020). The role of β-carotene and vitamin A in atherogenesis: Evidences from preclinical and clinical studies. Biochim. Biophys. Acta Mol. Cell Biol. Lipids.

[31] Csepanyi E., Czompa A., Szabados-Furjesi P., Lekli I., Balla J., Balla G., Tosaki A., Bak I. (2018). The effects of long-term, low-and high-dose beta-carotene treatment in Zucker diabetic fatty rats: The role of HO-1. Int. J. Mol. Sci..

[32] Stevens G.A., Bennett J.E., Hennocq Q., Lu Y., De-Regil L.M., Rogers L., Danaei G., Li G., White R.A., Flaxman S.R. (2015). Trends and mortality effects of vitamin A deficiency in children in 138 low-income and middle-income countries between 1991 and 2013: A pooled analysis of population-based surveys. Lancet Glob. Health.

[33] Imsic M., Winkler S., Tomkins B., Jones R. (2010). Effect of storage and cooking on β-carotene isomers in carrots (*Daucus carota* L. cv. ‘Stefano’). J. Agric. Food Chem..

[34] Knockaert G., Lemmens L., Van Buggenhout S., Hendrickx M., Van Loey A. (2012). Changes in β-carotene bioaccessibility and concentration during processing of carrot puree. Food Chem..

[35] Schieber A., Carle R. (2005). Occurrence of carotenoid cis-isomers in food: Technological, analytical, and nutritional implications. Trends Food Sci. Technol..

[36] Rodriguez-Amaya D.B. (2015). Food Carotenoids: Chemistry, Biology and Technology.

[37] Erdman J.W., Bierer T.L., Gugger E.T. (1993). Absorption and transport of carotenoids. Ann. N. Y. Acad. Sci..

[38] Nagarajaiah S.B., Prakash J. (2015). Nutritional composition, acceptability, and shelf stability of carrot pomace-incorporated cookies with special reference to total and β-carotene retention. Cogent Food Agric..

[39] Kaya-Celiker H., Mallikarjunan K. (2012). Better nutrients and therapeutics delivery in food through nanotechnology. Food Eng. Rev..

[40] Gul K., Tak A., Singh A., Singh P., Yousuf B., Wani A.A. (2015). Chemistry, encapsulation, and health benefits of β-carotene-A review. Cogent Food Agric..

[41] Teng Z., Xu R., Wang Q. (2015). Beta-lactoglobulin-based encapsulating systems as emerging bioavailability enhancers for nutraceuticals: A review. RSC Adv..

[42] Chen L., Bai G., Yang R., Zang J., Zhou T., Zhao G. (2014). Encapsulation of β-carotene within ferritin nanocages greatly increases its water-solubility and thermal stability. Food Chem..

[43] Gutiérrez F.J., Albillos S.M., Casas-Sanz E., Cruz Z., García-Estrada C., García-Guerra A., García-Reverter J., García-Suárez M., Gatón P., González-Ferrero C. (2013). Methods for the nanoencapsulation of β-carotene in the food sector. Trends Food Sci. Technol..

[44] Zhu F., Du B., Xu B. (2018). Anti-inflammatory effects of phytochemicals from fruits, vegetables, and food legumes: A review. Crit. Rev. Food Sci. Nutr..

[45] Boon C.S., McClements D.J., Weiss J., Decker E.A. (2010). Factors influencing the chemical stability of carotenoids in foods. Crit. Rev. Food Sci. Nutr..

[46] Dos Santos P.P., de Aguiar Andrade L., Flôres S.H., de Oliveira Rios A. (2018). Nanoencapsulation of carotenoids: A focus on different delivery systems and evaluation parameters. J. Food Sci. Technol..

[47] Rehman A., Tong Q., Jafari S.M., Assadpour E., Shehzad Q., Aadil R.M., Iqbal M.W., Rashed M.M.A., Mushtaq B.S., Ashraf W. (2020). Carotenoid-loaded nanocarriers: A comprehensive review. Adv. Colloid Interface Sci..

[48] Mao L., Wang D., Liu F., Gao Y. (2018). Emulsion design for the delivery of β-carotene in complex food systems. Crit. Rev. Food Sci. Nutr..

[49] Maurya V.K., Singh J., Ranjan V., Gothandam K.M., Bohn T., Pareek S. (2020). Factors affecting the fate of β-carotene in the human gastrointestinal tract: A narrative review. Int. J. Vitam. Nutr. Res..

[50] Parker R.S. (1996). Absorption, metabolism, and transport of carotenoids. FASEB J..

[51] Donhowe E.G., Kong F. (2014). Beta-carotene: Digestion, microencapsulation, and in vitro bioavailability. Food Bioprocess Technol..

[52] Van het Hof K.H., West C.E., Weststrate J.A., Hautvast J.G.A.J. (2000). Dietary factors that affect the bioavailability of carotenoids. J. Nutr..

[53] Iddir M., Degerli C., Dingeo G., Desmarchelier C., Schleeh T., Borel P., Larondelle Y., Bohn T. (2019). Whey protein isolate modulates beta-carotene bioaccessibility depending on gastro-intestinal digestion conditions. Food Chem..

[54] Schweiggert R.M., Mezger D., Schimpf F., Steingass C.B., Carle R. (2012). Influence of chromoplast morphology on carotenoid bioaccessibility of carrot, mango, papaya, and tomato. Food Chem..

[55] Hedren E., Diaz V., Svanberg U. (2002). Estimation of carotenoid accessibility from carrots determined by an in vitro digestion method. Eur. J. Clin. Nutr..

[56] Rock C.L., Lovalvo J.L., Emenhiser C., Ruffin M.T., Flatt S.W., Schwartz S.J. (1998). Bioavailability of β-carotene is lower in raw than in processed carrots and spinach in women. J. Nutr..

[57] Edwards A.J., Nguyen C.H., You C.-S., Swanson J.E., Emenhiser C., Parker R.S. (2002). α-and β-Carotene from a commercial carrot puree are more bioavailable to humans than from boiled-mashed carrots, as determined using an extrinsic stable isotope reference method. J. Nutr..

[58] Hornero-Méndez D., Mínguez-Mosquera M.I. (2007). Bioaccessibility of carotenes from carrots: Effect of cooking and addition of oil. Innov. Food Sci. Emerg. Technol..

[59] Hof K.H.V.H., de Boer B.C., Tijburg L.B., Lucius B.R., Zijp I., West C.E., Hautvast J.G., Weststrate J.A. (2000). Carotenoid bioavailability in humans from tomatoes processed in different ways determined from the carotenoid response in the triglyceride-rich lipoprotein fraction of plasma after a single consumption and in plasma after four days of consumption. J. Nutr..

[60] Lemmens L., Van Buggenhout S., Van Loey A.M., Hendrickx M.E. (2010). Particle size reduction leading to cell wall rupture is more important for the β-carotene bioaccessibility of raw compared to thermally processed carrots. J. Agric. Food Chem..

[61] Yonekura L., Nagao A. (2007). Intestinal absorption of dietary carotenoids. Mol. Nutr. Food Res..

[62] Faulks R.M., Southon S. (2005). Challenges to understanding and measuring carotenoid bioavailability. Biochim. Biophys. Acta Mol. Basis Dis..

[63] Rich G.T., Faulks R.M., Wickham M.S.J., Fillery-Travis A. (2003). Solubilization of carotenoids from carrot juice and spinach in lipid phases: II. Modeling the duodenal environment. Lipids.

[64] Rich G.T., Fillery-Travis A., Parker M.L. (1998). Low pH enhances the transfer of carotene from carrot juice to olive oil. Lipids.

[65] Iddir M., Dingeo G., Yaruro J.F.P., Hammaz F., Borel P., Schleeh T., Desmarchelier C., Larondelle Y., Bohn T. (2020). Influence of soy and whey protein, gelatin and sodium caseinate on carotenoid bioaccessibility. Food Funct..

[66] Bohn T. (2008). Bioavailability of non-provitamin A carotenoids. Curr. Nutr. Food Sci..

[67] Yeum K.-J., Russell R.M. (2002). Carotenoid bioavailability and bioconversion. Annu. Rev. Nutr..

[68] Fernández-García E., Carvajal-Lérida I., Jarén-Galán M., Garrido-Fernández J., Pérez-Gálvez A., Hornero-Méndez D. (2012). Carotenoids bioavailability from foods: From plant pigments to efficient biological activities. Food Res. Int..

[69] Roodenburg A.J.C., Leenen R., van het Hof K.H., Weststrate J.A., Tijburg L.B.M. (2000). Amount of fat in the diet affects bioavailability of lutein esters but not of α-carotene, β-carotene, and vitamin E in humans. Am. J. Clin. Nutr..

[70] Castenmiller J.J.M., West C.E., Linssen J.P.H., van het Hof K.H., Voragen A.G.J. (1999). The food matrix of spinach is a limiting factor in determining the bioavailability of β-carotene and to a lesser extent of lutein in humans. J. Nutr..

[71] Hollander D., Ruble P.E. (1978). beta-carotene intestinal absorption: Bile, fatty acid, pH, and flow rate effects on transport. Am. J. Physiol. Endoc. Metab..

[72] Corte-Real J., Iddir M., Soukoulis C., Richling E., Hoffmann L., Bohn T. (2016). Effect of divalent minerals on the bioaccessibility of pure carotenoids and on physical properties of gastro-intestinal fluids. Food Chem..

[73] Corte-Real J., Bertucci M., Soukoulis C., Desmarchelier C., Borel P., Richling E., Hoffmann L., Bohn T. (2017). Negative effects of divalent mineral cations on the bioaccessibility of carotenoids from plant food matrices and related physical properties of gastro-intestinal fluids. Food Funct..

[74] Prince M.R., Frisoli J.K. (1993). Beta-carotene accumulation in serum and skin. Am. J. Clin. Nutr..

[75] Unlu N.Z., Bohn T., Clinton S.K., Schwartz S.J. (2005). Carotenoid absorption from salad and salsa by humans is enhanced by the addition of avocado or avocado oil. J. Nutr..

[76] Jørgensen J.R., Fitch M.D., Mortensen P.B., Fleming S.E. (2001). In vivo absorption of medium-chain fatty acids by the rat colon exceeds that of short-chain fatty acids. Gastroenterology.

[77] Huo T., Ferruzzi M.G., Schwartz S.J., Failla M.L. (2007). Impact of fatty acyl composition and quantity of triglycerides on bioaccessibility of dietary carotenoids. J. Agric. Food Chem..

[78] Yonekura L., Nagao A. (2009). Soluble fibers inhibit carotenoid micellization in vitro and uptake by Caco-2 cells. Biosci. Biotechnol. Biochem..

[79] Rock C.L., Swendseid M.E. (1992). Plasma β-carotene response in humans after meals supplemented with dietary pectin. Am. J. Clin. Nutr..

[80] Riedl J., Linseisen J., Hoffmann J.R., Wolfram G.N. (1999). Some dietary fibers reduce the absorption of carotenoids in women. J. Nutr..

[81] Van Bennekum A., Werder M., Thuahnai S.T., Han C.-H., Duong P., Williams D.L., Wettstein P., Schulthess G., Phillips M.C., Hauser H. (2005). Class B scavenger receptor-mediated intestinal absorption of dietary β-carotene and cholesterol. Biochemistry.

[82] Cervantes-Paz B., de Jesús Ornelas-Paz J., Ruiz-Cruz S., Rios-Velasco C., Ibarra-Junquera V., Yahia E.M., Gardea-Béjar A.A. (2017). Effects of pectin on lipid digestion and possible implications for carotenoid bioavailability during pre-absorptive stages: A review. Food Res. Int..

[83] Bohn T., Desmarchelier C., Dragsted L.O., Nielsen C.S., Stahl W., Ruhl R., Keijer J., Borel P. (2017). Host-related factors explaining interindividual variability of carotenoid bioavailability and tissue concentrations in humans. Mol. Nutr. Food Res..

[84] Reboul E. (2013). Absorption of vitamin A and carotenoids by the enterocyte: Focus on transport proteins. Nutrients.

[85] Böhm V., Lietz G., Olmedilla-Alonso B., Phelan D., Reboul E., Bánati D., Borel P., Corte-Real J., de Lera A.R., Desmarchelier C. (2020). From carotenoid intake to carotenoid blood and tissue concentrations—Implications for dietary intake recommendations. Nutr. Rev..

[86] Mapelli-Brahm P., Margier M., Desmarchelier C., Halimi C., Nowicki M., Borel P., Meléndez-Martínez A.J., Reboul E. (2019). Comparison of the bioavailability and intestinal absorption sites of phytoene, phytofluene, lycopene and β-carotene. Food Chem..

[87] Roman M.J., Burri B.J., Singh R.P. (2012). Release and bioaccessibility of β-carotene from fortified almond butter during in vitro digestion. J. Agric. Food Chem..

[88] Garrett D.A., Failla M.L., Sarama R.J. (1999). Development of an in vitro digestion model for estimating the bioavailability of carotenoids from meals. J. Agric. Food Chem..

[89] Minekus M., Alminger M., Alvito P., Ballance S., Bohn T., Bourlieu C., Carriere F., Boutrou R., Corredig M., Dupont D. (2014). A standardised static in vitro digestion method suitable for food–an international consensus. Food Funct..

[90] Brodkorb A., Egger L., Alminger M., Alvito P., Assunção R., Ballance S., Bohn T., Bourlieu-Lacanal C., Boutrou R., Carrière F. (2019). INFOGEST static in vitro simulation of gastrointestinal food digestion. Nat. Protoc..

[91] Rao J., Decker E.A., Xiao H., McClements D.J. (2013). Nutraceutical nanoemulsions: Influence of carrier oil composition (digestible versus indigestible oil) on β-carotene bioavailability. J. Sci. Food Agric..

[92] Qian C., Decker E.A., Xiao H., McClements D.J. (2012). Nanoemulsion delivery systems: Influence of carrier oil on β-carotene bioaccessibility. Food Chem..

[93] Grune T., Lietz G., Palou A., Ross A.C., Stahl W., Tang G., Thurnham D., Yin S.A., Biesalski H.K. (2010). β-Carotene is an important vitamin A source for humans. J. Nutr..

[94] Fiedor J., Burda K. (2014). Potential role of carotenoids as antioxidants in human health and disease. Nutrients.

[95] Borba C.M., Tavares M.N., Macedo L.P., Araújo G.S., Furlong E.B., Dora C.L., Burkert J.F.M. (2019). Physical and chemical stability of β-carotene nanoemulsions during storage and thermal process. Food Res. Int..

[96] Borel P., Desmarchelier C. (2017). Genetic variations associated with vitamin A status and vitamin A bioavailability. Nutrients.

[97] Maurya V.K., Aggarwal M. (2017). Factors influencing the absorption of vitamin D in GIT: An overview. J. Food Sci. Technol..

[98] Aguilar-Pérez K.M., Avilés-Castrillo J.I., Medina D.I., Parra-Saldivar R., Iqbal H. (2020). Insight into nanoliposomes as smart nanocarriers for greening the twenty-first century biomedical settings. Front. Bioeng. Biotechnol..

[99] Soukoulis C., Bohn T. (2018). A comprehensive overview on the micro- and nano-technological encapsulation advances for enhancing the chemical stability and bioavailability of carotenoids. Crit. Rev. Food Sci. Nutr..

[100] Maurya V.K., Aggarwal M. (2017). Enhancing bio-availability of vitamin D by nano-engineered based delivery systems-An overview. Int. J. Curr. Microbiol. Appl. Sci..

[101] Maurya V.K., Bashir K., Aggarwal M. (2020). Vitamin D microencapsulation and fortification: Trends and technologies. J. Steroid Biochem. Mol. Biol..

[102] Rostamabadi H., Falsafi S.R., Jafari S.M. (2019). Nanoencapsulation of carotenoids within lipid-based nanocarriers. J. Control. Release.

[103] Maurya V.K., Aggarwal M., Ranjan V., Gothandam K.M. (2020). Improving bioavailability of vitamin A in food by encapsulation: An update. Nanoscience in Medicine Vol. 1.

[104] Matencio A., Navarro-Orcajada S., García-Carmona F., López-Nicolás J.M. (2020). Applications of cyclodextrins in food science. A review. Trends Food Sci. Technol..

[105] Crini G., Fourmentin S., Fenyvesi É., Torri G., Fourmentin M., Morin-Crini N. (2018). Fundamentals and applications of cyclodextrins. Cyclodextrin Fundamentals, Reactivity and Analysis.

[106] Arima H., Motoyama K., Higashi T. (2017). Potential use of cyclodextrins as drug carriers and active pharmaceutical ingredients. Chem. Pharm. Bull..

[107] Adeoye O., Cabral-Marques H. (2017). Cyclodextrin nanosystems in oral drug delivery: A mini review. Int. J. Pharm..

[108] Chilajwar S.V., Pednekar P.P., Jadhav K.R., Gupta G.J., Kadam V.J. (2014). Cyclodextrin-based nanosponges: A propitious platform for enhancing drug delivery. Expert Opin. Drug Deliv..

[109] Shafaei Z., Ghalandari B., Vaseghi A., Divsalar A., Haertlé T., Saboury A.A., Sawyer L. (2017). β-Lactoglobulin: An efficient nanocarrier for advanced delivery systems. Nanomed. NBM.

[110] Liu F., Ma C., Gao Y., McClements D.J. (2017). Food-grade covalent complexes and their application as nutraceutical delivery systems: A review. Compr. Rev. Food Sci..

[111] Chanphai P., Vesper A., Bariyanga J., Bérubé G., Tajmir-Riahi H. (2016). Review on the delivery of steroids by carrier proteins. J. Photochem. Photobiol. B Biol..

[112] Astray G., Mejuto J.C., Simal-Gandara J. (2020). Latest developments in the application of cyclodextrin host-guest complexes in beverage technology processes. Food Hydrocoll..

[113] Zaibunnisa A.H., Aini Marhanna M.N.A., AinunAtirah M. (2011). Characterisation and solubility study of γ-cyclodextrin and beta-carotene complex. Int. Food Res. J..

[114] Loftsson T., Brewster M.E., Masson M. (2004). Role of cyclodextrins in improving oral drug delivery. Am. J. Drug Deliv..

[115] Ozcelik B., Karadag A., Ersen S. Bioencapsulation of beta-carotene in three different methods. Proceedings of the XVIIth International Conference on Bioencapsulation.

[116] Letona C.A.M., Park C.S., Kim Y.R. (2017). Amylosucrase-mediated β-carotene encapsulation in amylose microparticles. Biotechnol. Prog..

[117] De Freitas Zômpero R.H., López-Rubio A., de Pinho S.C., Lagaron J.M., de la Torre L.G. (2015). Hybrid encapsulation structures based on β-carotene-loaded nanoliposomes within electrospun fibers. Colloids Surf. B.

[118] Liebler D.C., Stratton S.P., Kaysen K.L. (1997). Antioxidant actions of β-carotene in liposomal and microsomal membranes: Role of carotenoid-membrane incorporation and α-tocopherol. Arch. Biochem. Biophys..

[119] Carvalho J.M.P., Toniazzo T., Cavalcanti L.P., Moraes I.C.F., Oliveira C.L.P., Pinho S.C. (2015). Physico-chemical stability and structural characterization of thickened multilamellar beta-carotene-loaded liposome dispersions produced using a proliposome method. Colloid Polym. Sci..

[120] Bashiri S., Ghanbarzadeh B., Hamishehkar H., Dehghannia J. (2015). β-carotene loaded nanoliposome: Effects of gama–oryzanol on particle size stability and encapsulation. https://www.sid.ir/en/journal/ViewPaper.aspx?ID=502779.

[121] Schwartz J., Flynn E., Trickier D., Shklar G. (1991). Directed lysis of experimental cancer by β-carotene in liposomes. Nutr. Cancer.

[122] Silva C.R., Moraes M., Carvalho J.M.P., Pinho S.C. (2011). Characterization of Spray-Dried Phospholipid Particles for the Production of Beta-Carotene-Loaded Liposomes. https://www.semanticscholar.org/paper/Characterization-of-spray-dried-phospholipid-for-of-Silva-Moraes/a6bf4d0de1b7a9ec75b887a8d44b71480199a5ca.

[123] Tan C., Zhang Y., Abbas S., Feng B., Zhang X., Xia S. (2014). Modulation of the carotenoid bioaccessibility through liposomal encapsulation. Colloids Surf. B.

[124] Palozza P., Muzzalupo R., Trombino S., Valdannini A., Picci N. (2006). Solubilization and stabilization of β-carotene in niosomes: Delivery to cultured cells. Chem. Phys. Lipids.

[125] Nik A.M., Langmaid S., Wright A.J. (2012). Nonionic surfactant and interfacial structure impact crystallinity and stability of β-carotene loaded lipid nanodispersions. J. Agric. Food Chem..

[126] Yi J., Lam T.I., Yokoyama W., Cheng L.W., Zhong F. (2014). Cellular uptake of β-carotene from protein stabilized solid lipid nanoparticles prepared by homogenization–evaporation method. J. Agric. Food Chem..

[127] Helgason T., Awad T.S., Kristbergsson K., Decker E.A., McClements D.J., Weiss J. (2009). Impact of surfactant properties on oxidative stability of β-carotene encapsulated within solid lipid nanoparticles. J. Agric. Food Chem..

[128] Mehrad B., Ravanfar R., Licker J., Regenstein J.M., Abbaspourrad A. (2018). Enhancing the physicochemical stability of β-carotene solid lipid nanoparticle (SLNP) using whey protein isolate. Food Res. Int..

[129] Lima J.G.D., Brito-Oliveira T.C., Pinho S.C.D. (2016). Characterization and evaluation of sensory acceptability of ice creams incorporated with beta-carotene encapsulated in solid lipid microparticles. Food Sci. Technol..

[130] Gao S., McClements D.J. (2016). Formation and stability of solid lipid nanoparticles fabricated using phase inversion temperature method. Colloids Surf. A Physicochem. Eng. Asp..

[131] Gomes G.V.L., Simplício I.A.S., Souto E.B., Cardoso L.P., Pinho S.C. (2012). Development of a lipid particle for β-carotene encapsulation using a blend of tristearin and sunflower oil: Choice of lipid matrix and evaluation of shelf life of dispersions. Food Technol. Biotechnol..

[132] Qian C., Decker E.A., Xiao H., McClements D.J. (2013). Impact of lipid nanoparticle physical state on particle aggregation and β-carotene degradation: Potential limitations of solid lipid nanoparticles. Food Res. Int..

[133] Zhang L. (2013). Transparent Dispersions of Milk Fat-Based Solid Lipid Nanoparticles for Delivery of Beta-Carotene. Master’s Thesis.

[134] Gomes G.V.D.L., Borrin T.R., Cardoso L.P., Souto E., Pinho S.C.D. (2013). Characterization and shelf life of β-carotene loaded solid lipid microparticles produced with stearic acid and sunflower oil. Braz. Arch. Biol. Technol..

[135] Oliveira D.R.B., Michelon M., de Figueiredo Furtado G., Sinigaglia-Coimbra R., Cunha R.L. (2016). β-Carotene-loaded nanostructured lipid carriers produced by solvent displacement method. Food Res. Int..

[136] Hejri A., Khosravi A., Gharanjig K., Hejazi M. (2013). Optimisation of the formulation of β-carotene loaded nanostructured lipid carriers prepared by solvent diffusion method. Food Chem..

[137] Lacatusu I., Badea N., Ovidiu O., Bojin D., Meghea A. (2012). Highly antioxidant carotene-lipid nanocarriers: Synthesis and antibacterial activity. J. Nanopart. Res..

[138] Gomes G.V.L., Sola M.R., Marostegan L.F.P., Jange C.G., Cazado C.P.S., Pinheiro A.C., Vicente A.A., Pinho S.C. (2017). Physico-chemical stability and in vitro digestibility of beta-carotene-loaded lipid nanoparticles of cupuacu butter (*Theobroma grandiflorum*) produced by the phase inversion temperature (PIT) method. J. Food Eng..

[139] Ariviani S., Anggrahini S., Naruki S., Raharjo S. (2015). Characterization and chemical stability evaluation of β-carotene microemulsions prepared by spontaneous emulsification method using VCO and palm oil as oil phase. Int. Food Res. J..

[140] Mao Y., Dubot M., Xiao H., McClements D.J. (2013). Interfacial engineering using mixed protein systems: Emulsion-based delivery systems for encapsulation and stabilization of β-carotene. J. Agric. Food Chem..

[141] Neves M.A., Ribeiro H.S., Fujiu K.B., Kobayashi I., Nakajima M. (2008). Formulation of controlled size PUFA-loaded oil-in-water emulsions by microchannel emulsification using β-carotene-rich palm oil. Ind. Eng. Chem. Res..

[142] Salvia-Trujillo L., Qian C., Martín-Belloso O., McClements D.J. (2013). Influence of particle size on lipid digestion and β-carotene bioaccessibility in emulsions and nanoemulsions. Food Chem..

[143] Yao M., McClements D.J., Xiao H. (2015). Improving oral bioavailability of nutraceuticals by engineered nanoparticle-based delivery systems. Curr. Opin. Food Sci..

[144] Jo Y.-J., Kwon Y.-J. (2014). Characterization of β-carotene nanoemulsions prepared by microfluidization technique. Food Sci. Biotechnol..

[145] Campardelli R., Adami R., Reverchon E. (2012). Preparation of stable aqueous nanodispersions of β-carotene by supercritical assisted injection in a liquid antisolvent. Procedia Eng..

[146] Meroni E., Raikos V. (2018). Physicochemical stability, antioxidant properties and bioaccessibility of β-carotene in orange oil-in-water beverage emulsions: Influence of carrier oil types. Food Funct..

[147] Teixé-Roig J., Oms-Oliu G., Ballesté-Muñoz S., Odriozola-Serrano I., Martín-Belloso O. (2020). Improving the in vitro bioaccessibility of β-carotene using pectin added nanoemulsions. Foods.

[148] Zirak M.B., Pezeshki A. (2015). Effect of surfactant concentration on the particle size, stability and potential zeta of beta carotene nano lipid carrier. Int. J. Curr. Microbiol. App. Sci..

[149] Salvia-Trujillo L., Qian C., Martín-Belloso O., McClements D.J. (2013). Modulating β-carotene bioaccessibility by controlling oil composition and concentration in edible nanoemulsions. Food Chem..

[150] Chen H., Zhong Q. (2015). Thermal and UV stability of β-carotene dissolved in peppermint oil microemulsified by sunflower lecithin and Tween 20 blend. Food Chem..

[151] Qian C., Decker E.A., Xiao H., McClements D.J. (2012). Physical and chemical stability of β-carotene-enriched nanoemulsions: Influence of pH, ionic strength, temperature, and emulsifier type. Food Chem..

[152] Hasani F., Pezeshki A., Hamishehkar H. (2015). Effect of surfactant and oil type on size droplets of β-carotene-bearing nanoemulsions. Int. J. Curr. Microbiol. Appl. Sci..

[153] Muhamad I.I., Fen L.S., Hui N.H., Mustapha N.A. (2011). Genipin-cross-linked kappa-carrageenan/carboxymethyl cellulose beads and effects on beta-carotene release. Carbohydr. Polym..

[154] Liang R., Huang Q., Ma J., Shoemaker C.F., Zhong F. (2013). Effect of relative humidity on the store stability of spray-dried beta-carotene nanoemulsions. Food Hydrocoll..

[155] Liang R., Shoemaker C.F., Yang X., Zhong F., Huang Q. (2013). Stability and bioaccessibility of β-carotene in nanoemulsions stabilized by modified starches. J. Agric. Food Chem..

[156] Barragán P. (2013). Studies of Process Intensification for the Development of Hydrophilic and Hydrophobic β-Carotene Formulations. http://uvadoc.uva.es/handle/10324/4076.

[157] Liu F., Wang D., Sun C., Gao Y. (2016). Influence of polysaccharides on the physicochemical properties of lactoferrin–polyphenol conjugates coated β-carotene emulsions. Food Hydrocoll..

[158] Lin Q., Liang R., Ye A., Singh H., Zhong F. (2017). Effects of calcium on lipid digestion in nanoemulsions stabilized by modified starch: Implications for bioaccessibility of β-carotene. Food Hydrocoll..

[159] Chu B.S., Ichikawa S., Kanafusa S., Nakajima M. (2008). Stability of protein-stabilised β-carotene nanodispersions against heating, salts and pH. J. Sci. Food Agric..

[160] Sáiz-Abajo M.-J., González-Ferrero C., Moreno-Ruiz A., Romo-Hualde A., González-Navarro C.J. (2013). Thermal protection of β-carotene in re-assembled casein micelles during different processing technologies applied in food industry. Food Chem..

[161] Yi J., Fan Y., Zhang Y., Zhao L. (2016). Characterization of catechin-α-lactalbumin conjugates and the improvement in β-carotene retention in an oil-in-water nanoemulsion. Food Chem..

[162] Cornacchia L., Roos Y.H. (2011). State of dispersed lipid carrier and interface composition as determinants of beta-carotene stability in oil-in-water emulsions. J. Food Sci..

[163] Cornacchia L., Roos Y.H. (2011). Stability of β-carotene in protein-stabilized oil-in-water delivery systems. J. Agric. Food Chem..

[164] Salem A. (2015). Use of Whey Protein Nanoparticles for the Encapsulation and Sustained Delivery of Beta-Carotene and Zinc Micronutrient. Master’s Thesis.

[165] Yi J., Zhang Y., Liang R., Zhong F., Ma J. (2014). Beta-carotene chemical stability in nanoemulsions was improved by stabilized with beta-lactoglobulin–catechin conjugates through free radical method. J. Agric. Food Chem..

[166] Caliskan G., Lim A.S.L., Roos Y.H. (2015). Beta-carotene stability in extruded snacks produced using interface engineered emulsions. Int. J. Food Prop..

[167] Yi J., Li Y., Zhong F., Yokoyama W. (2014). The physicochemical stability and in vitro bioaccessibility of beta-carotene in oil-in-water sodium caseinate emulsions. Food Hydrocoll..

[168] Yin L.-J., Chu B.-S., Kobayashi I., Nakajima M. (2009). Performance of selected emulsifiers and their combinations in the preparation of β-carotene nanodispersions. Food Hydrocoll..

[169] Liu F., Wang D., Sun C., McClements D.J., Gao Y. (2016). Utilization of interfacial engineering to improve physicochemical stability of β-carotene emulsions: Multilayer coatings formed using protein and protein–polyphenol conjugates. Food Chem..

[170] Liu W., Wang J., McClements D.J., Zou L. (2018). Encapsulation of β-carotene-loaded oil droplets in caseinate/alginate microparticles: Enhancement of carotenoid stability and bioaccessibility. J. Funct. Foods.

[171] Yi J., Zhong F., Zhang Y., Yokoyama W., Zhao L. (2015). Effects of lipids on in vitro release and cellular uptake of β-carotene in nanoemulsion-based delivery systems. J. Agric. Food Chem..

[172] Salvia Trujillo L. (2014). Nanoemulsions as Delivery Systems of Food Ingredients: Improving Food Safety and Functionality. Ph.D. Thesis.

[173] Sutter S.C., Buera M.P., Elizalde B.E. (2007). β-Carotene encapsulation in a mannitol matrix as affected by divalent cations and phosphate anion. Int. J. Pharm..

[174] Chu B.-S., Ichikawa S., Kanafusa S., Nakajima M. (2007). Preparation and characterization of β-carotene nanodispersions prepared by solvent displacement technique. J. Agric. Food Chem..

[175] Yang Y., Guo Y., Sun R., Wang X. (2016). Self-assembly and β-carotene loading capacity of hydroxyethyl cellulose-graft-linoleic acid nanomicelles. Carbohydr. Polym..

[176] Moeller H., Martin D., Schrader K., Hoffmann W., Lorenzen P.C. (2017). Native casein micelles as nanocarriers for β-carotene: pH-and temperature-induced opening of the micellar structure. Int. J. Food Sci..

[177] Ge W., Li D., Chen M., Wang X., Liu S., Sun R. (2015). Characterization and antioxidant activity of β-carotene loaded chitosan-graft-poly(lactide) nanomicelles. Carbohydr. Polym..

[178] Roohinejad S., Oey I., Wen J., Lee S.J., Everett D.W., Burritt D.J. (2015). Formulation of oil-in-water β-carotene microemulsions: Effect of oil type and fatty acid chain length. Food Chem..

[179] Cao-Hoang L., Fougère R., Waché Y. (2011). Increase in stability and change in supramolecular structure of β-carotene through encapsulation into polylactic acid nanoparticles. Food Chem..

[180] Jain A., Sharma G., Kushwah V., Garg N.K., Kesharwani P., Ghoshal G., Singh B., Shivhare U.S., Jain S., Katare O.P. (2017). Methotrexate and beta-carotene loaded-lipid polymer hybrid nanoparticles: A preclinical study for breast cancer. Nanomedicine.

[181] Wang T., Wang R., Chen Z., Zhong Q. (2016). Coating oil droplets with rice proteins to control the release rate of encapsulated beta-carotene during in vitro digestion. RSC Adv..

[182] Chuacharoen T., Sabliov C.M. (2016). The potential of zein nanoparticles to protect entrapped β-carotene in the presence of milk under simulated gastrointestinal (GI) conditions. LWT—Food Sci. Technol..

[183] Lim A.S.L., Burdikova Z., Sheehan J.J., Roos Y.H. (2016). Carotenoid stability in high total solid spray dried emulsions with gum Arabic layered interface and trehalose–WPI composites as wall materials. Innov. Food Sci. Emerg. Technol..

[184] Kim J.-Y., Huber K.C. (2016). Preparation and characterization of corn starch-β-carotene composites. Carbohydr. Polym..

[185] Singh B.B., Shakil N.A., Kumar J., Walia S., Kar A. (2015). Development of slow release formulations of β-carotene employing amphiphilic polymers and their release kinetics study in water and different pH conditions. J. Food Sci. Technol..

[186] de Paz E., Martín Á., Bartolomé A., Largo M., Cocero M.J. (2014). Development of water-soluble β-carotene formulations by high-temperature, high-pressure emulsification and antisolvent precipitation. Food Hydrocoll..

[187] Yi J., Lam T.I., Yokoyama W., Cheng L.W., Zhong F. (2015). Beta-carotene encapsulated in food protein nanoparticles reduces peroxyl radical oxidation in Caco-2 cells. Food Hydrocoll..

[188] De Paz E., Martín Á., Cocero M.J. Production of water-soluble β-carotene formulations by high pressure processes. Proceedings of the III Iberoamerican Conference on Supercritical Fluids.

[189] Soukoulis C., Tsevdou M., Andre C.M., Cambier S., Yonekura L., Taoukis P.S., Hoffmann L. (2017). Modulation of chemical stability and in vitro bioaccessibility of beta-carotene loaded in kappa-carrageenan oil-in-gel emulsions. Food Chem..

[190] Fan Y., Yi J., Zhang Y., Wen Z., Zhao L. (2017). Physicochemical stability and in vitro bioaccessibility of β-carotene nanoemulsions stabilized with whey protein-dextran conjugates. Food Hydrocoll..

[191] Akinosho H.O., Wicker L. (2015). Stability of β-carotene loaded emulsions vary by viscosity of hydroxypropyl methylcellulose dispersions. LWT-Food Sci. Technol..

[192] Akrami M. (2016). Gum Arabic-Caseinate Nanocomplexes Bearing Beta-Carotene (1): Studying of Complex Formation by FTIR, DSC, Turbidity and Rheology. https://www.sid.ir/en/journal/ViewPaper.aspx?id=553536.

[193] Chen D., Zhao C.-X., Lagoin C., Hai M., Arriaga L.R., Koehler S., Abbaspourrad A., Weitz D.A. (2017). Dispersing hydrophobic natural colourant β-carotene in shellac particles for enhanced stability and tunable colour. R. Soc. Open Sci..

[194] Chen J., Li F., Li Z., McClements D.J., Xiao H. (2017). Encapsulation of carotenoids in emulsion-based delivery systems: Enhancement of β-carotene water-dispersibility and chemical stability. Food Hydrocoll..

[195] Chloe M.O., Davidovich-Pinhas M., Wright A.J., Barbut S., Marangoni A.G. (2017). Ethylcelluloseoleogels for lipophilic bioactive delivery–effect of oleogelation on in vitro bioaccessibility and stability of beta-carotene. Food Funct..

[196] Deng X.X., Zhang N., Tang C.H. (2017). Soy protein isolate as a nanocarrier for enhanced water dispersibility, stability and bioaccessibility of β-carotene. J. Sci. Food Agric..

[197] Dobrzanski C.D. (2016). Comparison of Non-Toxic Methods for Creating Beta-Carotene Encapsulated in Pmmananoparticles. https://rucore.libraries.rutgers.edu/rutgers-lib/51269/.

[198] Fernández A., Torres-Giner S., Lagarón J.M. Encapsulation of the functional ingredient beta-carotene in electrospun PLA fibers of interest in bioactive food packaging and food processing applications. Proceedings of the 23rd IAPRI Symposium of Packaging.

[199] Mahfoudhi N., Hamdi S. (2015). Kinetic degradation and storage stability of β-carotene encapsulated by freeze-drying using almond gum and gum Arabic as wall materials. J. Food Process. Preserv..

[200] Mahfoudhi N., Hamdi S. (2014). Kinetic degradation and storage stability of β-carotene encapsulated by spray drying using almond gum and gum arabic as wall materials. Polym. Eng. Sci..

[201] Hejri A., Gharanjig K., Khosravi A., Hejazi M. (2013). Effect of surfactants on kinetics of β-carotene photodegradation in emulsions. Chem. Eng. Commun..

[202] Sharif H.R., Goff H.D., Majeed H., Shamoon M., Liu F., Nsor-Atindana J., Haider J., Liang R., Zhong F. (2017). Physicochemical properties of β-carotene and eugenol co-encapsulated flax seed oil powders using OSA starches as wall material. Food Hydrocoll..

[203] Meng J., Kang T.-T., Wang H.-F., Zhao B.-B., Lu R.-R. (2018). Physicochemical properties of casein-dextran nanoparticles prepared by controlled dry and wet heating. Int. J. Biol. Macromol..

[204] Luo X., Zhou Y., Bai L., Liu F., Deng Y., McClements D.J. (2017). Fabrication of β-carotene nanoemulsion-based delivery systems using dual-channel microfluidization: Physical and chemical stability. J. Colloid Interface Sci..

[205] Fan Y., Gao L., Yi J., Zhang Y., Yokoyama W. (2017). Development of β-carotene loaded organogel based nanoemulsion with improved *invitro* and *in vivo* bioaccessibility. J. Agric. Food Chem..

[206] Lu W., Kelly A., Miao S. (2017). Bioaccessibility and cellular uptake of β-carotene encapsulated in model O/W emulsions: Influence of initial droplet size and emulsifiers. Nanomaterials.

[207] Zhang J., Zhang X., Wang X., Huang Y., Yang B., Pan X., Wu C. (2017). The influence of maltodextrin on the physicochemical properties and stabilization of beta-carotene emulsions. AAPS Pharm. Sci. Tech..

[208] Thais C., Oliveira B., Camila V., Samantha C. (2017). Encapsulation of beta carotene in lipid microparticles stabilized with hydrolyzed soy protein isolate: Production parameters, alphaâ tocopherol coencapsulation and stability under stress conditions. J. Food Sci..

[209] Ozvural E.B., Huang Q. (2017). Quality differences of hamburger patties incorporated with encapsulated β-carotene both as an additive and edible coating. J. Food Process. Preserv..

[210] Shao P., Qiu Q., Xiao J., Zhu Y., Sun P. (2017). Chemical stability and in vitro release properties of β-carotene in emulsions stabilized by *Ulvafasciata* polysaccharide. Int. J. Biol. Macromol..

[211] Wang M., Fu Y., Chen G., Shi Y., Li X., Zhang H., Shen Y. (2018). Fabrication and characterization of carboxymethyl chitosan and tea polyphenols coating on zein nanoparticles to encapsulate β-carotene by anti-solvent precipitation method. Food Hydrocoll..

[212] Sharif H.R., Goff H.D., Majeed H., Liu F., Nsor-Atindana J., Haider J., Liang R., Zhong F. (2017). Physicochemical stability of β-carotene and α-tocopherol enriched nanoemulsions: Influence of carrier oil, emulsifier and antioxidant. Colloids Surf. A Physicochem. Eng. Asp..

[213] Soukoulis C., Tsevdou M., Yonekura L., Cambier S., Taoukis P.S., Hoffmann L. (2017). Does kappa-carrageenan thermoreversible gelation affect β-carotene oxidative degradation and bioaccessibility in o/w emulsions?. Carbohydr. Polym..

[214] Thakur D., Jain A., Ghoshal G., Shivhare U.S., Katare O.P. (2017). Microencapsulation of β-carotene based on casein/guar gum blend using zeta potential-yield stress phenomenon: An approach to enhance photo-stability and retention of functionality. AAPS Pharm. Sci. Technol..

[215] Gu L., Su Y., Zhang M., Chang C., Li J., McClements D.J., Yang Y. (2017). Protection of β-carotene from chemical degradation in emulsion-based delivery systems using antioxidant interfacial complexes: Catechin-egg white protein conjugates. Food Res. Int..

[216] Xia Z., McClements D.J., Xiao H. (2017). Influence of lipid content in a corn oil preparation on the bioaccessibility of β-carotene: A comparison of low-fat and high-fat samples. J. Food Sci..

[217] Mun S., McClements D.J. (2017). Influence of simulated in-mouth processing (size reduction and alpha-amylase addition) on lipid digestion and β-carotene bioaccessibility in starch-based filled hydrogels. LWT-Food Sci. Technol..

[218] Morowvat M.H., Ghasemi Y. (2016). Spray-drying microencapsulation of β-carotene contents in powdered *Dunaliella salina* biomass. Int. J. Pharm. Clin. Res..

[219] Miyazawa T., Nakagawa K., Harigae T., Onuma R., Kimura F., Fujii T., Miyazawa T. (2015). Distribution of β-carotene-encapsulated polysorbate 80-coated poly (D, L-lactide-co-glycolide) nanoparticles in rodent tissues following intravenous administration. Int. J. Nanomed..

[220] Gupta S.S., Ghosh M. (2012). In vitro study of anti-oxidative effects of β-carotene and α-lipoic acid for nanocapsulated lipids. LWT-Food Sci. Technol..

[221] Hou Z., Zhang M., Liu B., Yan Q., Yuan F., Xu D., Gao Y. (2012). Effect of chitosan molecular weight on the stability and rheological properties of β-carotene emulsions stabilized by soybean soluble polysaccharides. Food Hydrocoll..

[222] Tavares J.K., de Souza A.A.U., de Oliveira J.V., Priamo W.L., de Souza S.M.A.G.U. (2016). Modeling of the controlled release of beta carotene into anhydrous ethanol from microcapsules. Open Nano.

[223] Wagner L.A., Warthesen J.J. (1995). Stability of spray-dried encapsulated carrot carotenes. J. Food Sci..

[224] Dluzewska E., Florowska A., Jasiorowska E. (2011). Effect of carrier type of stability of β-carotene micro-encapsulated using spray-drying method. Zywnosc Nauka Technol. Jakosc.

[225] Aissa A.F., Bianchi M.L.P., Ribeiro J.C., Hernandes L.C., de Faria A.F., Mercadante A.Z., Antunes L.M.G. (2012). Comparative study of β-carotene and microencapsulated β-carotene: Evaluation of their genotoxic and antigenotoxic effects. Food Chem. Toxicol..

[226] González-Reza R.M., Quintanar-Guerrero D., Flores-Minutti J.J., Gutiérrez-Cortez E., Zambrano-Zaragoza M.L. (2015). Nanocapsules of β-carotene: Thermal degradation kinetics in a scraped surface heat exchanger (SSHE). LWT—Food Sci. Technol..

[227] Jain A., Thakur D., Ghoshal G., Katare O.P., Shivhare U.S. (2016). Characterization of microcapsulated β-carotene formed by complex coacervation using casein and gum tragacanth. Int. J. Biol. Macromol..

[228] Donhowe E.G. (2013). Microencapsulation of β-Carotene: Characterization, In Vitro Release, and Bioavailability. Master’s Thesis.

[229] Nik A.M., Wright A.J., Corredig M. (2011). Micellization of beta-carotene from soy-protein stabilized oil-in-water emulsions under in vitro conditions of lipolysis. J. Am. Oil Chem. Soc..

[230] Franceschi E., De Cezaro A., Ferreira S.R.S., Kunita M.H., Muniz E.C., Rubira A.F., Oliveira J. (2010). Co-precipitation of beta-carotene and bio-polymer using supercritical carbon dioxide as antisolvent. Open Chem. Eng. J..

[231] Priamo W.L., de Cezaro A.M., Ferreira S.R.S., Oliveira J.V. (2010). Precipitation and encapsulation of β-carotene in PHBV using carbon dioxide as anti-solvent. J. Supercrit. Fluids.

[232] Divya P., Anbarasan B., Ramaprabhu S. Preparation and characterization of beta-carotene encapsulated chitosan, oleic acid coated Fe_3_O_4_nanoparticles. Proceedings of the International Conference on Nanoscience, Technology and Societal Implications (NSTSI).

[233] Kim J.-Y., Seo T.-R., Lim S.-T. (2013). Preparation of aqueous dispersion of β-carotene nano-composites through complex formation with starch dextrin. Food Hydrocoll..

[234] Ramoneda X.A., Ponce-Cevallos P.A., Buera M.D.P., Elizalde B.E. (2011). Degradation of β-carotene in amorphous polymer matrices. Effect of water sorption properties and physical state. J. Sci. Food Agric..

[235] Silva H.D., Cerqueira M.A., Souza B.W.S., Ribeiro C., Avides M.C., Quintas M.A.C., Coimbra J.S.R., Carneiro-da-Cunha M.G., Vicente A.A. (2011). Nanoemulsions of β-carotene using a high-energy emulsification–evaporation technique. J. Food Eng..

[236] Trentin A., De Lamo S., Güell C., López F., Ferrando M. (2011). Protein-stabilized emulsions containing beta-carotene produced by premix membrane emulsification. J. Food Eng..

[237] Qi J. (2004). Microencapsulation of Beta-Carotene in Pea Protein Wall System.

[238] Barreto H.R.D. (2008). Physical Stability of a Lactose-Trehalose Matrix for Nano-Encapsulation of Beta-Carotene by Spray Drying.

[239] Silva M.M., Nora L., Cantillano R.F.F., Paese K., Guterres S.S., Pohlmann A.R., Costa T.M.H., de Oliveira Rios A. (2016). The production, characterization, and the stability of carotenoids loaded in lipid-core nanocapsules. Food Bioprocess Technol..

[240] Gupta S.S., Ghosh M. (2015). Synthesis, characterization, stability evaluation and release kinetics of fiber-encapsulated carotene nano-capsules. Grasas Aceites.

[241] Peinado I., Mason M., Romano A., Biasioli F., Scampicchio M. (2016). Stability of β-carotene in polyethylene oxide electrospun nanofibers. Appl. Surf. Sci..

[242] Donhowe E.G., Flores F.P., Kerr W.L., Wicker L., Kong F. (2014). Characterization and in vitro bioavailability of β-carotene: Effects of microencapsulation method and food matrix. LWT—Food Sci. Technol..

[243] Zhang Z., Zhang R., McClements D.J. (2016). Encapsulation of β-carotene in alginate-based hydrogel beads: Impact on physicochemical stability and bioaccessibility. Food Hydrocoll..

[244] Park S., Mun S., Kim Y.-R. (2018). Effect of xanthan gum on lipid digestion and bioaccessibility of β-carotene-loaded rice starch-based filled hydrogels. Food Res. Int..

[245] O’Sullivan C. (2016). In-Vitro Bioaccessibility and Stability of Beta-Carotene in Ethylcelluloseoleogels. http://hdl.handle.net/10214/9716.

[246] Shao Y., Tang C.-H. (2016). Gel-like pea protein pickering emulsions at pH3.0 as a potential intestine-targeted and sustained-release delivery system for β-carotene. Food Res. Int..

[247] Soukoulis C., Cambier S., Hoffmann L., Bohn T. (2016). Chemical stability and bioaccessibility of β-carotene encapsulated in sodium alginate o/w emulsions: Impact of Ca2+ mediated gelation. Food Hydrocoll..

[248] Wang S., Chen X., Shi M., Zhao L., Li W., Chen Y., Lu M., Wu J., Yuan Q., Li Y. (2015). Absorption of whey protein isolated (WPI)-stabilized β-carotene emulsions by oppositely charged oxidized starch microgels. Food Res. Int..

[249] Liu F., Tang C.-H. (2016). Soy glycinin as food-grade pickering stabilizers: Part. III. Fabrication of gel-like emulsions and their potential as sustained-release delivery systems for β-carotene. Food Hydrocoll..

[250] Mun S., Kim Y.-R., McClements D.J. (2015). Control of β-carotene bioaccessibility using starch-based filled hydrogels. Food Chem..

[251] Kong L., Bhosale R., Ziegler G.R. (2018). Encapsulation and stabilization of β-carotene by amylose inclusion complexes. Food Res. Int..

[252] Ramezanli T., Kilfoyle B.E., Zhang Z., Michniak-Kohn B.B. (2017). Polymeric nanospheres for topical delivery of vitamin D3. Int. J. Pharm..

[253] Uyen N.T.T., Hamid Z.A.A., Tram N.X.T., Ahmad N. (2020). Fabrication of alginate microspheres for drug delivery: A review. Int. J. Biol. Macromol..

[254] Lengyel M., Kállai-Szabó N., Antal V., Laki A.J., Antal I. (2019). Microparticles, microspheres, and microcapsules for advanced drug delivery. Sci. Pharm..

[255] Cascone S., Lamberti G. (2020). Hydrogel-based commercial products for biomedical applications: A review. Int. J. Pharm..

[256] Batista R.A., Espitia P.J.P., Quintans J.D.S.S., Freitas M.M., Cerqueira M.Â., Teixeira J.A., Cardoso J.C. (2019). Hydrogel as an alternative structure for food packaging systems. Carbohydr. Polym..

[257] Qu B., Luo Y. (2020). Chitosan-based hydrogel beads: Preparations, modifications and applications in food and agriculture sectors–A review. Int. J. Biol. Macromol..

[258] Huynh P.T. (2012). Solvent-Free Beta-Carotene Nanoparticles for Food Fortification.

[259] Nascimento L.G.L., Casanova F., Silva N.F.N., de Carvalho Teixeira A.V.N., de Carvalho A.F. (2020). Casein-based hydrogels: A mini-review. Food Chem..

[260] Klein M., Poverenov E. (2020). Natural biopolymer-based hydrogels for use in food and agriculture. J. Sci. Food Agric..

[261] Zheng H., Mao L., Cui M., Liu J., Gao Y. (2020). Development of food-grade bigels based on κ-carrageenan hydrogel and monoglyceride oleogels as carriers for β-carotene: Roles of oleogel fraction. Food Hydrocoll..

[262] Li J., Jia X., Yin L. (2021). Hydrogel: Diversity of structures and applications in food science. Food Rev. Int..

[263] Chen X., Liang R., Zhong F., Yokoyama W.H. (2020). Effect of β-carotene status in microcapsules on its in vivo bioefficacy and in vitro bioaccessibility. Food Hydrocoll..

[264] Chen X., Liang R., Zhong F., Ma J., John N.-A., Goff H.D., Yokoyama W.H. (2019). Effect of high concentrated sucrose on the stability of OSA-starch-based β-carotene microcapsules. Food Hydrocoll..

[265] Erdoğar N., Akkın S., Bilensoy E. (2018). Nanocapsules for drug delivery: An updated review of the last decade. Recent Pat. Drug Deliv. Formul..

[266] Vijeth S., Heggannavar G.B., Kariduraganavar M.Y. (2019). Encapsulating wall materials for micro-/nanocapsules. Microencapsulation-Processes, Technologies and Industrial Applications.

[267] Amgoth C., Kumar K., Medhi H., Paik P. (2014). Polymeric nanocapsules for drug delivery applications. Nanotechnol. Anim. Health Prod..

[268] Zhao L., Duan G., Zhang G., Yang H., He S., Jiang S. (2020). Electrospun functional materials toward food packaging applications: A review. Nanomaterials.

[269] De Farias B.S., Junior T.R.S.A.C., de Almeida Pinto L.A. (2019). Chitosan-functionalized nanofibers: A comprehensive review on challenges and prospects for food applications. Int. J. Biol. Macromol..

[270] Kumar T.S.M., Kumar K.S., Rajini N., Siengchin S., Ayrilmis N., Rajulu A.V. (2019). A comprehensive review of electrospun nanofibers: Food and packaging perspective. Compos. B Eng..

[271] Zhang C., Li Y., Wang P., Zhang H. (2020). Electrospinning of nanofibers: Potentials and perspectives for active food packaging. Compr. Rev. Food Sci..

[272] Rezaei A., Nasirpour A., Fathi M. (2015). Application of cellulosic nanofibers in food science using electrospinning and its potential risk. Compr. Rev. Food Sci..

[273] Li X., Lin L., Zhu Y., Liu W., Yu T., Ge M. (2013). Preparation of ultrafine fast-dissolving cholecalciferol-loaded poly (vinyl pyrrolidone) fiber mats via electrospinning. Polym. Compos..

[274] Barroso L., Viegas C., Vieira J., Pego C., Costa J., Fonte P. (2020). Lipid-based carriers for food ingredients delivery. J. Food. Eng..

[275] Chaudhari V.S., Murty U.S., Banerjee S. (2020). Lipidic nanomaterials to deliver natural compounds against cancer: A review. Environ. Chem. Lett..

[276] Sarkar A., Mackie A.R. (2020). Engineering oral delivery of hydrophobic bioactives in real world scenarios. Curr. Opin. Colloid Interface Sci..

[277] Shukla D., Chakraborty S., Singh S., Mishra B. (2011). Lipid-based oral multiparticulate formulations–advantages, technological advances and industrial applications. Expert Opin. Drug Deliv..

[278] Mozafari M.R., Flanagan J., Matia-Merino L., Awati A., Omri A., Suntres Z.E., Singh H. (2006). Recent trends in the lipid-based nanoencapsulation of antioxidants and their role in foods. J. Sci. Food Agric..

[279] Akhavan S., Assadpour E., Katouzian I., Jafari S.M. (2018). Lipid nano scale cargos for the protection and delivery of food bioactive ingredients and nutraceuticals. Trends Food Sci. Technol..

[280] de Souza Simões L., Madalena D.A., Pinheiro A.C., Teixeira J.A., Vicente A.A., Ramos O.L. (2017). Micro-and nano bio-based delivery systems for food applications: In vitro behavior. Adv. Colloid Interface Sci..

[281] Fathi M., Mozafari M.R., Mohebbi M. (2012). Nanoencapsulation of food ingredients using lipid based delivery systems. Trends Food Sci. Technol..

[282] Sun X., Bandara N. (2019). Applications of reverse micelles technique in food science: A comprehensive review. Trends Food Sci. Technol..

[283] Kiss É. (2020). Nanotechnology in food systems: A review. Acta Aliment..

[284] Wakaskar R.R. (2017). General overview of lipid-polymer hybrid nanoparticles, dendrimers, micelles, liposomes, spongosomes and cubosomes. J. Drug Target..

[285] Keskin D., Tezcaner A. (2017). Micelles as delivery system for cancer treatment. Curr. Pharm. Des..

[286] Tanbour R., Martins A.M., Pitt W.G., Husseini G.A. (2016). Drug delivery systems based on polymeric micelles and ultrasound: A review. Curr. Pharm. Des..

[287] Gothwal A., Khan I., Gupta U. (2016). Polymeric micelles: Recent advancements in the delivery of anticancer drugs. Pharm. Res..

[288] Ranadheera C.S., Liyanaarachchi W.S., Chandrapala J., Dissanayake M., Vasiljevic T. (2016). Utilizing unique properties of caseins and the casein micelle for delivery of sensitive food ingredients and bioactives. Trends Food Sci. Technol..

[289] Kim S., Shi Y., Kim J.Y., Park K., Cheng J.-X. (2010). Overcoming the barriers in micellar drug delivery: Loading efficiency, in vivo stability, and micelle–cell interaction. Expert Opin. Drug Deliv..

[290] McClements D.J., Xiao H. (2017). Is nano safe in foods? Establishing the factors impacting the gastrointestinal fate and toxicity of organic and inorganic food-grade nanoparticles. NPJ Sci. Food.

[291] Du Y., Bao C., Huang J., Jiang P., Jiao L., Ren F., Li Y. (2019). Improved stability, epithelial permeability and cellular antioxidant activity of β-carotene via encapsulation by self-assembled α-lactalbumin micelles. Food Chem..

[292] Fernandez P., André V., Rieger J., Kühnle A. (2004). Nano-emulsion formation by emulsion phase inversion. Colloids Surf. A Physico Chem..

[293] Flores-Miranda G.A., del Toro G.V., Yánez-Fernández J. (2015). Stability evaluation of β-carotene nanoemulsions prepared by homogenization-emulsification process using stearic acid as oil phase. Rev. Mex. Ing. Quim..

[294] Xia Z., Han Y., Du H., McClements D.J., Tang Z., Xiao H. (2020). Exploring the effects of carrier oil type on in vitro bioavailability of β-carotene: A cell culture study of carotenoid-enriched nanoemulsions. LWT-Food Sci. Technol..

[295] Gomes M., Santos D.T., Meireles M.A.A. (2012). Trends in particle formation of bioactive compounds using supercritical fluids and nanoemulsions. Food Public Health.

[296] Zhang R., Zhang Z., Kumosani T., Khoja S., Abualnaja K.O., McClements D.J. (2016). Encapsulation of β-carotene in nanoemulsion-based delivery systems formed by spontaneous emulsification: Influence of lipid composition on stability and bioaccessibility. Food Biophys..

[297] Rao J., McClements D.J. (2010). Stabilization of phase inversion temperature nanoemulsions by surfactant displacement. J. Agric. Food Chem..

[298] McClements D.J. (2012). Nanoemulsions versus microemulsions: Terminology, differences, and similarities. Soft Matter.

[299] Meng Q., Long P., Zhou J., Ho C.-T., Zou X., Chen B., Zhang L. (2019). Improved absorption of β-carotene by encapsulation in an oil-in-water nanoemulsion containing tea polyphenols in the aqueous phase. Food Res. Int..

[300] NishitaniYukuyama M., Tomiko Myiake Kato E., Lobenberg R., AraciBou-Chacra N. (2017). Challenges and future prospects of nanoemulsion as a drug delivery system. Curr. Pharm. Des..

[301] Choi S.J., McClements D.J. (2020). Nanoemulsions as delivery systems for lipophilic nutraceuticals: Strategies for improving their formulation, stability, functionality and bioavailability. Food Sci. Biotechnol..

[302] Daeihamed M., Dadashzadeh S., Haeri A., Faghih Akhlaghi M. (2017). Potential of liposomes for enhancement of oral drug absorption. Curr. Drug Deliv..

[303] Huang Z., Li X., Zhang T., Song Y., She Z., Li J., Deng Y. (2014). Progress involving new techniques for liposome preparation. Asian J. Pharm. Sci..

[304] Bozzuto G., Molinari A. (2015). Liposomes as nanomedical devices. Int. J. Nanomed..

[305] Nekkanti V., Venkatesan N., Betageri G. (2015). Proliposomes for oral delivery: Progress and challenges. Curr. Pharm. Biotechnol..

[306] Liu W., Ye A., Singh H., Sagis L.M.C. (2015). Progress in applications of liposomes in food systems. Microencapsulation and Microspheres for Food Applications.

[307] Kim J.-S. (2016). Liposomal drug delivery system. J. Pharm. Sci..

[308] Mozafari M.R., Khosravi-Darani K., Borazan G.G., Cui J., Pardakhty A., Yurdugul S. (2008). Encapsulation of food ingredients using nanoliposome technology. Int. J. Food Prop..

[309] Amoabediny G., Haghiralsadat F., Naderinezhad S., Helder M.N., AkhoundiKharanaghi E., MohammadnejadArough J., Zandieh-Doulabi B. (2018). Overview of preparation methods of polymeric and lipid-based (noisome, solid lipid, liposome) nanoparticles: A comprehensive review. Int. J. Polym. Mater..

[310] Filipczak N., Pan J., Yalamarty S.S.K., Torchilin V.P. (2020). Recent advancements in liposome technology. Adv. Drug Deliv. Rev..

[311] Srinivasan V., Chavan S., Jain U., Tarwadi K. (2019). Liposomes for Nanodelivery Systems in Food Products. Nanoscience for Sustainable Agriculture.

[312] Liu X., Wang P., Zou Y.-X., Luo Z.-G., Tamer T.M. (2020). Co-encapsulation of vitamin C and β-carotene in liposomes: Storage stability, antioxidant activity, and in vitro gastrointestinal digestion. Food Res. Int..

[313] Tan C., Xue J., Abbas S., Feng B., Zhang X., Xia S. (2014). Liposome as a delivery system for carotenoids: Comparative antioxidant activity of carotenoids as measured by ferric reducing antioxidant power, DPPH assay and lipid peroxidation. J. Agric. Food Chem..

[314] Rashidinejad A., Birch E.J., Sun-Waterhouse D., Everett D.W. (2014). Delivery of green tea catechin and epigallocatechin gallate in liposomes incorporated into low-fat hard cheese. Food Chem..

[315] Elkholy N.S., Shafaa M.W., Mohammed H.S. (2020). Biophysical characterization of lutein or beta carotene-loaded cationic liposomes. RSC Adv..

[316] Payne N.I., Ambrose C.V., Timmins P., Ward M.D., Ridgway F. (1986). Proliposomes: A novel solution to an old problem. J. Pharm. Sci..

[317] Chordiya D., Shilpi S., Choudhary D., Saraogi G.K., Sharma M., Kalyane D., Tekade R.K. (2020). Proliposomes: A potential colloidal carrier for drug delivery applications. The Future of Pharmaceutical Product Development and Research.

[318] Khorasani S., Danaei M., Mozafari M.R. (2018). Nanoliposome technology for the food and nutraceutical industries. Trends Food Sci. Technol..

[319] Matos M., Pando D., Gutiérrez G. (2019). Nanoencapsulation of food ingredients by niosomes. Lipid-Based Nanostructures for Food Encapsulation Purposes.

[320] Wen J., Al Gailani M., Yin N., Rashidinejad A. (2018). Liposomes and niosomes. Liposomes and Niosomes Emulsion-Based Systems for Delivery of Food Active Compounds.

[321] Roohinejad S., Greiner R., Oey I., Wen J. (2018). Emulsion-Based Systems for Delivery of Food Active Compounds: Formation, Application, Health and Safety.

[322] Moghassemi S., Hadjizadeh A. (2014). Nano-niosomes as nanoscale drug delivery systems: An illustrated review. J. Control. Release.

[323] Arora R. (2007). Advances in niosome as a drug carrier: A review. Asian J. Pharm..

[324] Yasam V.R., Jakki S.L., Natarajan J., Kuppusamy G. (2014). A review on novel vesicular drug delivery: Proniosomes. Drug Deliv..

[325] Paliwal R., Paliwal S.R., Kenwat R., Kurmi B.D., Sahu M.K. (2020). Solid lipid nanoparticles: A review on recent perspectives and patents. Expert Opin. Ther. Pat..

[326] Ghanbarzadeh B., Keivani F., Mohammadi M. (2019). Encapsulation of food ingredients by solid lipid nanoparticles (SLNs). Lipid-Based Nanostructures for Food Encapsulation Purposes.

[327] Duong V.A., Nguyen T.T.L., Maeng H.J. (2020). Preparation of solid lipid nanoparticles and nanostructured lipid carriers for drug delivery and the effects of preparation parameters of solvent injection method. Molecules.

[328] Aditya N.P., Ko S. (2015). Solid lipid nanoparticles (SLNs): Delivery vehicles for food bioactives. RSC Adv..

[329] Trotta M., Debernardi F., Caputo O. (2003). Preparation of solid lipid nanoparticles by a solvent emulsification–diffusion technique. Int. J. Pharm..

[330] Trucillo P., Campardelli R. (2019). Production of solid lipid nanoparticles with a supercritical fluid assisted process. J. Supercrit. Fluids.

[331] Zardini A.A., Mohebbi M., Farhoosh R., Bolurian S. (2018). Production and characterization of nanostructured lipid carriers and solid lipid nanoparticles containing lycopene for food fortification. J. Food Sci. Technol..

[332] Salminen H., Ankenbrand J., Zeeb B., Bönisch G.B., Schäfer C., Kohlus R., Weiss J. (2019). Influence of spray drying on the stability of food-grade solid lipid nanoparticles. Food Res. Int..

[333] Da Silva Santos V., Ribeiro A.P.B., Santana M.H.A. (2019). Solid lipid nanoparticles as carriers for lipophilic compounds for applications in foods. Food Res. Int..

[334] Tamjidi F., Shahedi M., Varshosaz J., Nasirpour A. (2013). Nanostructured lipid carriers (NLC): A potential delivery system for bioactive food molecules. Innov. Food Sci. Emerg. Technol..

[335] Haider M., Abdin S.M., Kamal L., Orive G. (2020). Nanostructured lipid carriers for delivery of chemotherapeutics: A review. Pharmaceutics.

[336] Can Q., Johan C., Charlotta T. (2009). Carotenoids particle formation by supercritical fluid technologies. Chin. J. Chem. Eng..

[337] Subramaniam B., Siddik Z.H., Nagoor N.H. (2020). Optimization of nanostructured lipid carriers: understanding the types, designs, and parameters in the process of formulations. J. Nanopart. Res..

[338] Gomes G.V.L., Sola M.R., Rochetti A.L., Fukumasu H., Vicente A.A., Pinho S.C. (2019). β-carotene and α-tocopherol coencapsulated in nanostructured lipid carriers of murumuru (*Astrocaryum murumuru*) butter produced by phase inversio n temperature method: Characterisation, dynamic in vitro digestion and cell viability study. J. Microencapsul..

[339] Maurya V.K., Aggarwal M. (2019). A phase inversion based nanoemulsion fabrication process to encapsulate vitamin D3 for food applications. J. Steroid Biochem..

[340] Hentschel A., Gramdorf S., Müller R.H., Kurz T. (2008). β-Carotene-loaded nanostructured lipid carriers. J. Food Sci..

[341] Pezeshki A., Hamishehkar H., Ghanbarzadeh B., Fathollahy I., Nahr F.K., Heshmati M.K., Mohammadi M. (2019). Nanostructured lipid carriers as a favorable delivery system for β-carotene. Food Biosci..

[342] Azar F.A.N., Pezeshki A., Ghanbarzadeh B., Hamishehkar H., Mohammadi M. (2020). Nanostructured lipid carriers: Promising delivery systems for encapsulation of food ingredients. J. Sci. Food Agric..

[343] Gasa-Falcon A., Odriozola-Serrano I., Oms-Oliu G., Martín-Belloso O. (2020). Nanostructured lipid-based delivery systems as a strategy to increase functionality of bioactive compounds. Foods.

[344] Tamjidi F., Shahedi M., Varshosaz J., Nasirpour A. (2014). EDTA and α-tocopherol improve the chemical stability of astaxanthin loaded into nanostructured lipid carriers. Eur. J. Lipid Sci. Technol..

[345] Salminen H., Aulbach S., Leuenberger B.H., Tedeschi C., Weiss J. (2014). Influence of surfactant composition on physical and oxidative stability of quillaja saponin-stabilized lipid particles with encapsulated ω-3 fish oil. Colloids Surf. B.

[346] Yang C., Yan H., Jiang X., Xu H., Tsao R., Zhang L. (2020). Preparation of 9 Z-β-carotene and 9 Z-β-carotene high-loaded nanostructured lipid carriers: Characterization and storage stability. J. Agric. Food Chem..

[347] Dima C., Assadpour E., Dima S., Jafari S.M. (2020). Bioavailability of nutraceuticals: Role of the food matrix, processing conditions, the gastrointestinal tract, and nanodelivery systems. Compr. Rev. Food Sci..

[348] Dowling A.P. (2004). Development of nanotechnologies. Mater. Today.

[349] Chau C.-F., Wu S.-H., Yen G.-C. (2007). The development of regulations for food nanotechnology. Trends Food Sci. Technol..

[350] Amenta V., Aschberger K., Arena M., Bouwmeester H., Moniz F.B., Brandhoff P., Gottardo S., Marvin H.J., Mech A., Pesudo L.Q. (2015). Regulatory aspects of nanotechnology in the agri/feed/food sector in EU and non-EU countries. Regul. Toxicol. Pharmacol..

[351] Committee E.S. (2011). Guidance on the risk assessment of the application of nanoscience and nanotechnologies in the food and feed chain. EFSA J..

[352] Livney Y.D. (2015). Nanostructured delivery systems in food: Latest developments and potential future directions. Curr. Opin. Food Sci..

[353] Hardy A., Benford D., Halldorsson T., Jeger M.J., Knutsen H.K., More S., Naegeli H., Noteborn H., Ockleford C., Ricci A. (2018). Guidance on risk assessment of the application of nanoscience and nanotechnologies in the food and feed chain: Part 1, human and animal health. EFSA J..

[354] Akbari-Alavijeh S., Shaddel R., Jafari S.M. (2020). In vivo assays for evaluating the release of nanoencapsulated food ingredients. Release and Bioavailability of Nanoencapsulated Food Ingredients.

[355] Luo Y., Wang Q., Zhang Y. (2020). Biopolymer-based nanotechnology approaches to deliver bioactive compounds for food applications: A perspective on the past, present, and future. J. Agric. Food Chem..

[356] Müllertz A., Ogbonna A., Ren S., Rades T. (2010). New perspectives on lipid and surfactant based drug delivery systems for oral delivery of poorly soluble drugs. J. Pharm. Pharmacol..

[357] Santos D.T., Meireles M.A. (2010). Carotenoid pigments encapsulation: Fundamentals, techniques and recent trends. Open Chem. Eng. J..

[358] Salvia-Trujillo L., Verkempinck S., Rijal S.K., Van Loey A., Grauwet T., Hendrickx M. (2019). Lipid nanoparticles with fats or oils containing β-carotene: Storage stability and in vitro digestibility kinetics. Food Chem..

[359] Zambrano-Zaragoza M.L., Quintanar-Guerrero D., Del Real A., Piñon-Segundo E., Zambrano-Zaragoza J.F. (2017). The release kinetics of β-carotene nanocapsules/xanthan gum coating and quality changes in fresh-cut melon (cantaloupe). Carbohydr. Polym..

[360] Yang Y., McClements D.J. (2013). Vitamin E bioaccessibility: Influence of carrier oil type on digestion and release of emulsified α-tocopherol acetate. Food Chem..

[361] Pouton C.W., Porter C.J. (2008). Formulation of lipid-based delivery systems for oral administration: Materials, methods and strategies. Adv. Drug Deliv. Rev..

[362] Yáñez J.A., Wang S.W., Knemeyer I.W., Wirth M.A., Alton K.B. (2011). Intestinal lymphatic transport for drug delivery. Adv. Drug Deliv. Rev..

[363] Yao M., Chen J., Zheng J., Song M., McClements D.J., Xiao H. (2013). Enhanced lymphatic transport of bioactive lipids: Cell culture study of polymethoxyflavone incorporation into chylomicrons. Food Funct..

[364] McClements D.J. (2013). Edible lipid nanoparticles: Digestion, absorption, and potential toxicity. Prog. Lipid Res..

[365] Borel T., Sabliov C.M. (2014). Nanodelivery of bioactive components for food applications: Types of delivery systems, properties, and their effect on ADME profiles and toxicity of nanoparticles. Annu. Rev. Food Sci. Technol..

[366] Ozturk B., Argin S., Ozilgen M., McClements D.J. (2015). Nanoemulsion delivery systems for oil-soluble vitamins: Influence of carrier oil type on lipid digestion and vitamin D3 bioaccessibility. Food Chem..

